# Effects of circuit training or a nutritional intervention on body mass index and other cardiometabolic outcomes in children and adolescents with overweight or obesity

**DOI:** 10.1371/journal.pone.0245875

**Published:** 2021-01-28

**Authors:** Young-Gyun Seo, Hyunjung Lim, YoonMyung Kim, Young-Su Ju, Yong-jun Choi, Hye-Ja Lee, Han Byul Jang, Sang Ick Park, Kyung Hee Park

**Affiliations:** 1 Department of Family Medicine, Hallym University Sacred Heart Hospital, Anyang, Gyeonggi-do, Republic of Korea; 2 Department of Medical Nutrition, Kyung Hee University, Yongin, Gyeonggi-do, Republic of Korea; 3 University College, Yonsei University International Campus, Incheon, Republic of Korea; 4 Department of Occupational and Environmental Medicine, National Medical Center, Seoul, Republic of Korea; 5 Department of Social and Preventive Medicine & Health Services Research Center, Hallym University College of Medicine, Chucheon, Gangwon-do, Republic of Korea; 6 Center for Biomedical Sciences, Korea National Institute of Health, Cheongju, Chungbuk, Republic of Korea; University of California Los Angeles, UNITED STATES

## Abstract

**Objective:**

We aimed to assess the effectiveness of the first 6 months of a 24 month multidisciplinary intervention program including circuit training and a balanced diet in children and adolescents with obesity.

**Methods:**

A quasi-experimental intervention trial included 242 participants (age [mean±standard deviation]: 11.3±2.06 years, 97 girls) of at least 85th percentile of age- and sex-specific body mass index (BMI). Participants were grouped into three to receive usual care (usual care group), exercise intervention with circuit training (exercise group), or intensive nutritional and feedback intervention with a balanced diet (nutritional group). Primary outcome was BMI z-score, while secondary outcomes included body composition, cardiometabolic risk markers, nutrition, and physical fitness.

**Results:**

Among the participants, 80.6% had a BMI ≥ the 97th percentile for age and sex. The BMI z-score of the overall completers decreased by about 0.080 after 6 months of intervention (p < 0.001). After the intervention, both exercise and nutritional groups had significantly lower BMI z-scores than the baseline data by about 0.14 and 0.075, respectively (p < 0.05). Significant group by time interaction effects were observed between exercise versus usual care group in BMI z-score (β, -0.11; 95% confidence interval (CI), -0.20 to -0.023) and adiponectin (β, 1.31; 95% CI, 1.08 to 1.58); and between nutritional versus usual care group in waist circumference (β, -3.47; 95% CI, -6.06 to -0.89). No statistically significant differences were observed in any of the other secondary outcomes assessed.

**Conclusion:**

Multidisciplinary intervention including circuit training and a balanced diet for children and adolescents with obesity reduced the BMI z-score and improved cardiometabolic risk markers such as adiponectin and waist circumference.

## Introduction

Obesity in childhood, a serious health issue, reduces the quality of life and increases health care costs [1]. The overall rising trend in childhood and adolescence body mass index (BMI) has plateaued in many high-income countries, albeit at high levels, but has accelerated in parts of Asia [2]. Obesity in childhood increases the risk of adult obesity, which is associated with many health problems [3, 4]. It is also associated with cardiovascular risk, insulin resistance, type 2 diabetes, metabolic syndrome, and nonalcoholic fatty liver disease [5]. Therefore, to address these long-term health problems, it is necessary to develop effective multidisciplinary interventions for children and adolescents.

Many previous studies have evaluated the effects of bariatric surgery, drugs, or inpatient treatment for children with severe obesity. A systematic review revealed low to moderate evidence for a substantial reduction in BMI in adolescents with severe obesity following metabolic and bariatric surgery (after 5 years of follow-up), and very low to low evidence in the resolution of related co-morbidities [6]. Most studies reported weight regain within 1 to 12 years of follow-up. Other reviews and randomized clinical trials (RCTs) of pharmacologic intervention and inpatient treatment for children and adolescents with severe obesity reveal a controversial effect size for weight reduction, and intervention effects were not sustained in the long-term [7–9]. Surgery is invasive, drugs that are available to children are limited, and inpatient treatment is difficult to apply in real-world settings.

A recent network meta-analysis indicated that the most effective intervention in decreasing weight in children with overweight or obesity is ‘exercise intervention without parent’. In addition, ‘exercise intervention without parent’, ‘diet intervention with parent’, and ‘diet, exercise, and lifestyle intervention with parent’ were significantly effective treatments for children with obesity when compared with no intervention [10]. Previous studies reported positive effects of circuit training-based exercise interventions on obesity in children and adolescents [11, 12]. In addition, recent studies reported positive effects of balanced diet-based nutritional interventions on obesity in children and adolescents [13, 14]. Therefore, this study aimed to assess the effectiveness of the first 6 months of a 24 month multidisciplinary intervention program with circuit training and a balanced diet considered sustainable in a real-world setting with practical applicable as an intervention for those with both moderate and severe obesity.

## Materials and methods

### Study design and participants

Overall, 242 participants aged 6 to 17 years (145 boys and 97 girls) with ≥ 85th percentile of age- and sex-specific BMI according to the 2007 Korean National Growth Charts [15] were recruited into the long-term Intervention for Childhood and Adolescents Obesity via Activity and Nutrition (ICAAN) study through an online recruitment notice on websites and social networking services, television, newspapers, and flyers. Overweight was defined as a BMI ≥ 85th percentile for age and sex, mild to moderate obesity was defined as BMI ≥ 95th percentile for age and sex or ≥ 25 kg/m^2^, and severe obesity was defined as BMI ≥ 35 kg/m^2^ or ≥ 120% of the 95th percentile [15, 16].

**Fig 1:**
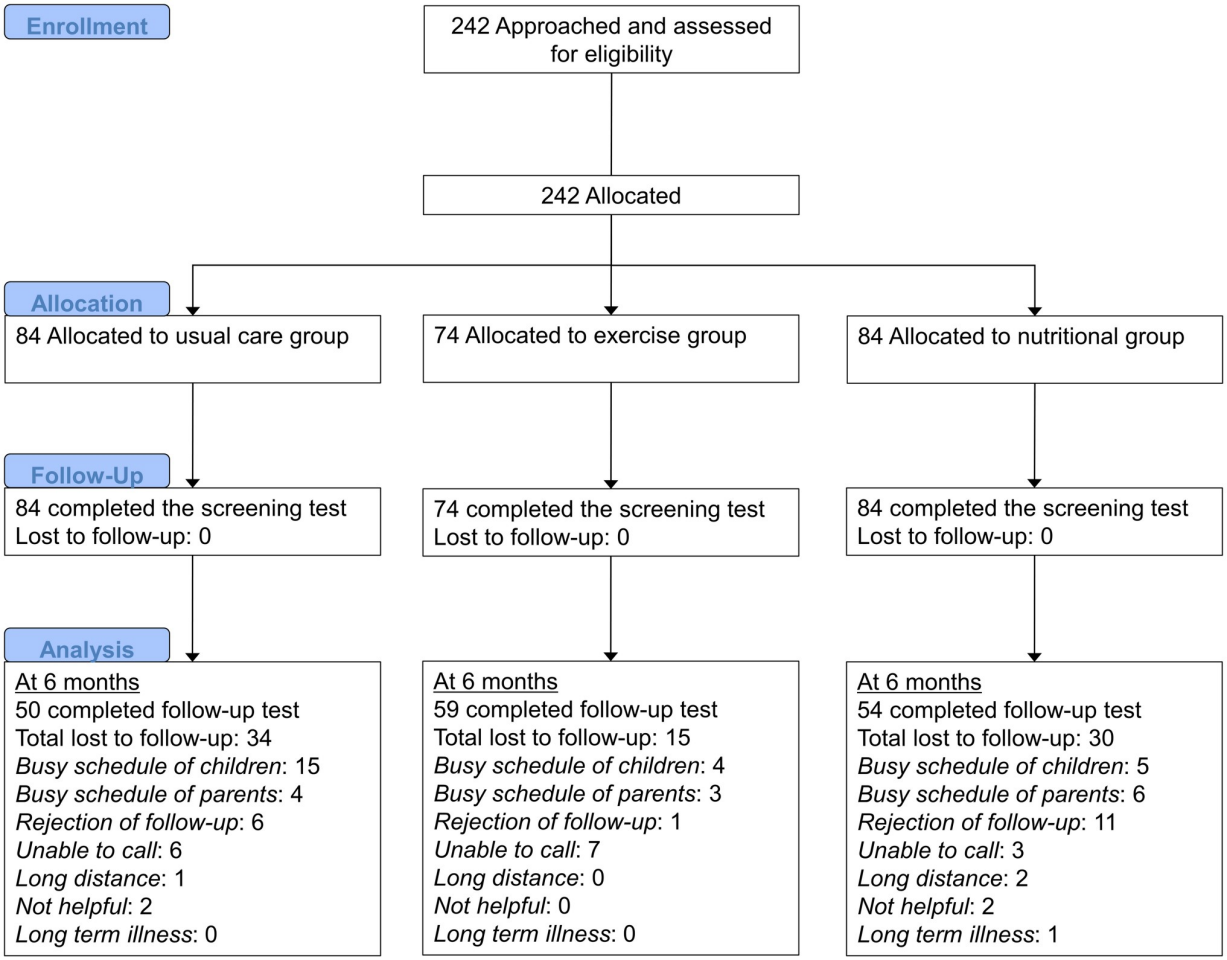
CONSORT flowchart.

The ICAAN study is a quasi-experimental intervention trial designed to test an intervention capable of preventing excessive weight gain and improving several health indices for children and adolescents with obesity in Korea. The ICAAN has three active treatment groups receiving a two-year intervention as follows: usual care for obesity (counseling on diet, physical activity, and behavioral modification; “usual care group”); an exercise intervention (“exercise group”); and an intensive nutritional and feedback intervention (“nutritional group”) group. The participants flowchart is shown in Fig 1. All 242 participants that were approached and assessed for eligibility met the criteria and agreed to participate. Although this study is not an RCT, we have tried to assign participants using a consecutive randomization procedure; however, geographic reasons and personal circumstances were also considered. The senior researcher (KHP), who was not blinded to the research aims, performed the allocation.

This study was conducted according to the guidelines laid down in the Declaration of Helsinki, and all procedures involving human participants were approved by the Hallym University Sacred Heart Hospital’s Institutional Review Board (approval number: 2016-I135). Written informed consent was obtained from all participants and their parents or caregivers. This study was registered at cris.nih.go.kr (identifier: KCT0002718).

A priori protocol was not published. The study protocols are shown in the S1 File (Korean) and the S2 File (English). The protocol was written prior to the start of the study and no changes were made after intervention commencement. Only the study registration has been delayed. The authors confirm that all ongoing and related trials for this intervention are registered.

Date at which the ethics committee approved the study: October 11, 2016.

Date range for patient recruitment and 6-month follow-up: November 26, 2016-February 10, 2018.

### Intervention

S1 Fig shows the flow chart of the intervention. ICAAN is being conducted in four phases over a 2-year period. Phases 1, 2, 3, and 4 comprise the intensive intervention (for 6 months), group activity (for 6 months), booster (for 3 months), and group activity (for 9 months), respectively.

All participants received the usual care, including one-to-one medical consultation, workbook provision for goal setting and behavioral modification, exercise counseling, physical activity monitoring and feedback, and one-to-one nutritional counseling. In addition, risk factors related to obesity as well as factors related to obesity management were selected and named Mission 5, and all participants were asked to participate. The contents of Mission 5 were as follows: drink water instead of other drinks; exercise more than 1 hour per day; reduce screen time to less than 2 hours per day; eat 5 or more vegetables and fruits every day; get enough sleep for more than 8 hours per day. At the first visit, a doctor explained anthropometric measurements and laboratory test results and assessed health risks and lifestyle of all participants. Every 6 months, the doctor reviewed the workbook and conducted one-to-one medical consultation. The contents of the workbook are shown in S1 Table. In addition to the medical consultation, all participants received exercise counseling from an exercise specialist, which focused on increasing and decreasing physical activity and inactivity, respectively. One-to-one nutritional counseling was conducted monthly by a clinical dietitian for all participants during phase 1. Nutrition counseling was based on a balanced diet that included sufficient water intake, recommended caloric intake and balanced distribution of nutrients [17, 18]. The contents of the nutritional counseling comprised of 8 sessions of 25 minutes each during phase 1 and are shown in S2 Table.

The participants assigned to the exercise group also received the usual care during the intervention period, and additionally participated in the exercise programs. The exercise program required participants to exercise 3 days/week for 60 minutes/session (one group exercise session and two home-based exercise sessions) at 60% to 80% of the maximal heart rate (HR). The group exercise program involved bodyweight circuit training, which consisted of 6 different exercises as shown in S3 Table. Each exercise was performed for 1 minute with 30 seconds rest; the entire workout took approximately 10 minutes to complete and was named the “ICAAN Exercise”. During the group exercise, the participants performed 4 to 5 sets of the ICAAN Exercise under the supervision of our health trainer. In order to ensure and maintain appropriate levels of exercise intensity during the group exercise, all participants wore a HR monitor (Polar H7 Bluetooth HR Sensor, Polar, Finland). All group exercise sessions were performed in the gym at the Hallym University Sacred Heart Hospital. The home-based exercise was a repeat process of the group exercise. During the home-based exercise, participants were asked to perform regular exercise at least 2 times per week for 60 minutes per session, consisting of 30 minutes of the ICAAN Exercise learned at the group exercise session and 30 minutes of regular aerobic exercise such as running and/or cycling. For the home-based exercise, a daily exercise journal prepared by the exercise group was reviewed every week for the first 3 months, and every other week for the next 3 months.

The participants assigned to the nutritional group also received the usual care during the intervention period. In addition, one-to-one customized nutritional counseling according to the Nutritional Care Process (NCP) model [19] was conducted monthly by a clinical dietitian during phase 1. The NCP contains four distinct but interrelated steps: nutritional assessment, diagnosis, intervention, and monitoring / evaluation, which enables systematic and efficient nutritional counseling. The nutritional counseling was comprised of 8 sessions during phase 1 and are shown in S2 Table. Each session was conducted for 25 minutes. The nutritional group was also provided with weekly nutritional feedback during phase 1.

### Questionnaire

Questionnaires completed by children and adolescents included dietary habits, physical activity (the Global Physical Activity Questionnaire [20, 21]), drinking, smoking, sleeping time, screen time (television / computer use), inactivity time, and mental health (depression, stress, etc.). Questionnaires completed by parents or caregivers included education, monthly household income, past medical history, living with both parents, and the child’s birth-related variables (birth weight, etc.).

### Dietary assessment

Dietary intakes were collected using 3-day food records (2 weekdays and 1 weekend day), after which, a clinical dietitian double-checked the records using food models. The participant nutrient intakes were assessed using a computer-aided nutritional analysis program (CAN Pro, Web version 5.0; The Korean Nutrition Society, Seoul, Korea, 2016).

### Anthropometric measurements and body composition

Body weight was measured by Bioelectrical Impedance Analysis (BIA) (InBody 720 Body Composition Analyzer, BioSpace Co., Ltd., Seoul, Korea) [22] after a 10-hour fast and voiding, with the participant barefoot wearing lightweight indoor clothing. Height was measured by a stadiometer (DS-103, DongSahn Jenix, Seoul, Korea) while the participant was barefoot. Weight and height were measured to the nearest 0.1 kg and 0.1 cm, respectively. BMI values (weight in kilograms divided by height in meters squared) were converted into percentiles and z-scores based on age- and sex-specific BMI of the 2007 Korean National Growth Charts [15]. Waist circumference (WC) was measured at midpoint between the last rib and the top of the iliac crest to the nearest 0.1 cm using a non-elastic tape measure. Parental measurement data (height, body weight, body composition, and WC) were also collected. To assess body composition, an additional whole-body dual-energy X-ray absorptiometry (DXA) (Lunar Prodigy Advance with pediatric software version enCore 14.0, GE Medical Systems Lunar, Madison, WI, USA) scanner performed a series of transverse one-centimeter scans starting at the participant’s head and progressing toward the feet after a 10-hour fast and voiding. DXA assessments were made by a certified radiology technologist. The values measured in the DXA were used for body composition analysis.

### Blood pressure and blood samples

The participants were seated in a quiet room and blood pressure (BP) measurements started after a 5–10 minutes rest using a digital automatic BP monitor (HEM-907, Omron, Kyoto, Japan) [23] in the morning. BP was measured twice in the right arm of the participants with a cuff adapted to the arm circumference; the mean value was used. Venous blood samples were obtained in the morning after a 12-hour overnight fast to determine the fasting plasma glucose (FPG), fasting plasma insulin, total cholesterol (TC), high-density lipoprotein cholesterol (HDL-C), low-density lipoprotein cholesterol (LDL-C), triglyceride (TG), aspartate aminotransferase (AST), alanine aminotransferase (ALT), gamma-glutamyl transferase (GGT), high-sensitivity C-reactive protein (CRP), and adiponectin. FPG was measured using UV assay with hexokinase (Cobas 8000 C702, Roche, Mannheim, Germany). Insulin was measured using electro-chemiluminescence immunoassay (Cobas 8000 e802, Roche, Mannheim, Germany). HDL-C and LDL-C were measured using homogeneous enzymatic colorimetric test (Cobas 8000 C702, Roche, Mannheim, Germany). TC, TG, AST, ALT, and GGT were measured using enzymatic assay (Cobas 8000 C702, Roche, Mannheim, Germany). CRP was measured using turbidimetric immunoassay (Cobas 8000 C702, Roche, Mannheim, Germany). Adiponectin was measured using enzyme-linked immunosorbent assay (VersaMax ELISA Microplate Reader, Molecular Devices, San Jose, CA, USA). The homeostasis model assessment for insulin resistance (HOMA-IR) was used to determine insulin sensitivity and was calculated using the following formula: [FPG Level (mg/dL) × Fasting Plasma Insulin Level (μU/mL)] / 405.

### Fitness test

Cardiorespiratory fitness was assessed via 3-minute YMCA step test because it is easy to perform and well tolerated by school-aged children [24, 25]. Prior to the test, the participants were asked to sit in a chair for 2 to 3 minutes of rest and instruction. During the test, the participants stepped (up-up, down-down) for 3 minutes on a 30 cm height step box. A metronome was used to ensure the step frequency (24 steps/minute) and set at 96 beats/minute. HR was recorded during the test and recovery phases. Post-exercise HR was measured for 1 minute after the end of the test. Muscular strength was assessed using 1-repetition maximal (1-RM) test before and after the intervention. Upper- and lower-body muscular strength were assessed using chest press and leg extension machines (Technogym, Italy), respectively. Prior to the test, participants were instructed by the exercise physiologists on the proper lifting techniques and test procedure, as prescribed by the American College of Sports Medicine’s guidelines [26]. Briefly, a perceived maximum weight was obtained from a participant following a warm-up of 5 to 10 repetitions at 40% to 60% of the perceived maximum weight and a subsequent test of 4 to 5 repetitions at 60% to 80% of their perceived maximum weight. Finally, a small amount of weight (2 to 5 kg) was added, and a 1-RM lift was attempted. If the lift was successful, the participant rested for about 3 minutes and then attempted to lift a heavier weight (additional 2 to 5 kg). If the lift was not successful, a small amount of weight was removed, and another 1-RM was attempted. The goal of the procedure was to determine the 1-RM within 4 maximal attempts.

### Outcomes

The primary outcome of the study was the BMI z-score (standardized using age- and sex-specific BMI from the 2007 Korean National Growth Charts [15]), and the percentage of the 95th percentile of age- and sex-specific BMI (%BMI_p95th_) [27]. Secondary outcomes included (1) body composition variables (body fat (BF), lean body mass (LM), etc.); (2) cardiometabolic risk markers (BP, FPG, insulin, HOMA-IR, TC, HDL-C, LDL-C, TG, AST, ALT, GGT, CRP, adiponectin, etc.); (3) nutrition (total energy intake); and (4) cardiorespiratory fitness and muscular strength. All questionnaires and tests were performed at baseline and 6 months.

### Sample size estimation

We calculated a sample size of 69 participants with 23 per group to detect the effects of the intervention on BMI z-score (80% power with a two-sided significance level set at 0.05). The estimated minimum clinically important differences between interventions and usual care were 0.1 for changes in BMI z-score [12, 14, 28]. The estimate for the standard deviation (SD) was chosen as 0.1 for changes in BMI z-score. To allow for 30% dropout, a sample size of 99 was sufficient to assess the effect of the intervention throughout the study period.

### Statistical analysis

The Shapiro-Wilk test was used to evaluate normality of the data [29]. The analysis was conducted by transforming the data to a logarithmic scale to achieve a bell-shaped (approximately normal) distribution of the required variables. We compared the baseline characteristics of the three treatment groups using the one-way analysis of variance (ANOVA) test for independent samples (after checking for the normal distributions of continuous variables). In addition, we used the Pearson’s chi-squared test for categorical variables. The paired t-test was used to compare the pre- and post-intervention outcomes (baseline and at 6 months). Mixed effects linear regression models for repeated measures were used to analyze between-group differences in outcomes over time. Intercept was used for the mixed model random effects at the individual level. We performed regression analyses after adjustment for confounding variables including age, sex, parental obesity, parental education, monthly household income, living with both parents, and sleep time. We also analyzed our data using the intention-to-treat (ITT) approach with the last observation carried forward method when data were missing.

All statistical analyses were conducted using Stata/MP, version 14.0 (StataCorp, College Station, TX, USA). All statistical tests were two-sided, and statistical significance was determined by p < 0.05.

## Results

### Baseline characteristics

Of 242 participants, 40.1% were girls, 32.6% had severe obesity, 80.6% had BMI ≥ 97th percentile for age and sex, and mean age of 11.3±2.06 years. No significant differences occurred in the proportion of severe obesity (28.6% vs. 39.2% vs. 31.0%, p = 0.34) and of BMI ≥ 97th percentile for age and sex (78.6% vs. 85.1% vs. 78.6%, p = 0.49) among the usual care, exercise, and nutritional group participants, respectively, at baseline. After 6 months of intervention, 163 participants (67.4%) were followed-up. No significant differences occurred in all characteristics and outcome variables between the participants who were followed-up and dropped out, at baseline (S4 and S5 Tables).

The participants with both baseline and 6-month follow-up data were defined as the completers, and their data were analyzed. Table 1 shows the baseline demographic characteristics and anthropometric measurements of completers. There were no significant differences in characteristics between the usual care, exercise, and nutritional groups at baseline. Table 2 shows the baseline laboratory test results, lifestyle measurements, and fitness test results of all participants and completers. There were also no significant differences between the three groups at baseline.

**Table 1:** Baseline demographic characteristics and anthropometric measurements of completers.

Characteristic	Completers			p-value
	Usual care group (n = 50)	Exercise group (n = 59)	Nutritional group (n = 54)	
**Age, years**	11.3±2.00	11.0±2.28	11.4±2.11	0.58
**Age, years**				0.16
6–9	12 (24.0)	24 (40.7)	17 (31.5)	
10–14	37 (74.0)	32 (54.2)	32 (59.3)	
15–17	1 (2.0)	3 (5.1)	5 (9.3)	
**Sex, No. (%)**				0.16
Male	30 (60.0)	30 (50.8)	37 (68.5)	
Female	20 (40.0)	29 (49.2)	17 (31.5)	
**Parental obesity, No. (%) (n = 43 / 52 / 50)**				0.11
None	6 (14.0)	7 (13.5)	14 (28.0)	
Either	37 (86.0)	45 (86.5)	36 (72.0)	
**Parental CVD history, No. (%) (n = 39 / 50 / 51)**				0.37
None	16 (41.0)	22 (44.0)	28 (54.9)	
Either	23 (59.0)	28 (56.0)	23 (45.1)	
**Parental education, No. (%) (n = 44 / 55 / 48)**				0.92
< College (both)	10 (22.7)	11 (20.0)	11 (22.9)	
≥ College (either)	34 (77.3)	44 (80.0)	37 (77.1)	
**Monthly household income, No. (%) (n = 48 / 59 / 51)**				0.77
< 3 million KRW	6 (12.5)	11 (18.6)	9 (17.6)	
3–5 million KRW	18 (37.5)	25 (42.4)	22 (43.1)	
≥ 5 million KRW	24 (50.0)	23 (39.0)	20 (39.2)	
**Living with both parents, No. (%) (n = 48 / 59 / 51)**				0.54
Yes	44 (91.7)	50 (84.7)	44 (86.3)	
No	4 (8.33)	9 (15.3)	7 (13.7)	
**Birth weight, kg (n = 49 / 55 / 50)**	3.33±0.52	3.41±0.43	3.36±0.52	0.66
**Body weight, kg**	66.7±19.3	65.2±16.8	68.4±19.0	0.65
**BMI, kg/m**^**2**^	28.2±4.09	28.5±4.35	28.6±4.32	0.91
**BMI z-score**	2.27±0.51	2.39±0.52	2.27±0.48	0.34
**%BMI**_**p95th**_**, %**^a^	115±1.13	118±1.13	115±1.12	0.38
**Waist circumference, cm**	87.2±11.9	86.9±10.7	89.9±10.4	0.29
**Body fat, kg**	27.9±8.58	27.1±7.96	28.5±8.99	0.68
**Body fat, %**	42.1±3.92	41.8±4.67	41.8±3.70	0.91
**Lean mass, kg**	36.5±10.8	35.7±9.33	37.5±9.67	0.62
**SBP, mmHg**	119±12.2	119±13.3	120±15.9	0.88
**DBP, mmHg**	68.8±8.74	67.8±10.3	69.5±9.61	0.65

Abbreviations: CVD, cardiovascular disease; KRW, Korean Republic Won; BMI, body mass index; %BMI_p95th_, percentage of the 95th percentile of age- and sex-specific body mass index; SBP, systolic blood pressure; DBP, diastolic blood pressure.

Data are presented as mean±standard deviation for continuous variables (one-way analysis of variance test) and number (%) for categorical variables (χ^2^ test). Percentages have been rounded up and may not total to 100.

^a^Geometric mean±standard deviation.

**Table 2:** Baseline laboratory test results, lifestyle measurements, and fitness test results of completers.

Characteristic	Completers			p-value
	Usual care group (n = 50)	Exercise group (n = 59)	Nutritional group (n = 54)	
**HOMA-IR**^a^	3.79±1.70	4.06±1.80	3.98±1.50	0.78
**TC, mg/dL**	178±26.6	178±25.2	170±28.6	0.39
**HDL-C, mg/dL**	50.6±10.8	51.4±12.5	48.7±9.82	0.43
**LDL-C, mg/dL**	115±23.0	112±23.2	110±25.9	0.58
**TG, mg/dL**^a^	97.0±1.62	102±1.51	104±1.64	0.72
**AST, U/L**^a^	23.0±1.56	23.4±1.57	26.1±1.61	0.32
**ALT, U/L**^a^	22.2±1.97	23.9±2.17	29.5±2.29	0.14
**GGT, U/L**^a^	19.4±1.41	20.1±1.52	23.6±1.72	0.061
**CRP, mg/L**^a^	1.36±2.10	1.42±2.40	1.79±2.18	0.18
**Adiponectin, μg/mL**^a^	8.25±1.45	7.00±1.51	8.15±1.55	0.061
**Total energy intake, kcal**^a^	2112±1.30	2089±1.26	2205±1.23	0.45
**Sleep time, hours (n = 48 / 53 / 50)**	8.69±3.28	8.06±2.35	8.44±2.25	0.48
**Inactivity time, hours (n = 43 / 46 / 50)**^a^	2.27±1.83	2.67±2.01	2.47±1.90	0.50
**Activity level, MET-minutes/week (n = 42 / 47 / 47)**^a^	1749±3.55	1657±2.58	2655±2.84	0.077
**Step test, post exam HR, BPM (n = 49 / 58 / 53)**	112±17.8	117±16.3	112±16.0	0.28
**Chest press, 1-RM, kg (n = 50 / 59 / 54)**	28.8±10.7	29.4±11.7	29.5±11.3	0.93
**Leg extension, 1-RM, kg (n = 50 / 58 / 54)**	43.4±18.6	44.1±19.4	44.8±18.4	0.93

Abbreviations: HOMA-IR, homeostasis model assessment for insulin resistance; TC, total cholesterol; HDL-C, high-density lipoprotein cholesterol; LDL-C, low-density lipoprotein cholesterol; TG, triglyceride; AST, aspartate aminotransferase; ALT, alanine aminotransferase; GGT, gamma-glutamyl transferase; CRP, high-sensitivity C-reactive protein; MET, metabolic equivalents (1 MET: oxygen consumption of 3.5 mL/kg/minute); HR, heart rate; BPM, beats per minute; RM, repetition maximum.

HOMA-IR = (Fasting Plasma Glucose Level (mg/dL) × Fasting Plasma Insulin Level (μU/mL)) / 405.

Data are expressed as mean±standard deviation unless otherwise indicated.

^a^Geometric mean±standard deviation.

### Within-group changes in the primary outcome

The mean BMI z-score of the completers showed a statistically significant decrease after 6 months of intervention (2.31±0.51 at baseline vs. 2.23±0.55 at 6-month follow-up, p < 0.001). At this time, the exercise group had significantly lower BMI z-scores (2.25±0.56 vs. 2.39±0.52, p < 0.001) and %BMI_p95th_ (114±1.13% vs. 118±1.13%, p < 0.001), and the nutritional group also had significantly lower BMI z-scores (2.19±0.55 vs. 2.27±0.48, p = 0.02) and %BMI_p95th_ (113±1.14% vs. 115±1.12%, p = 0.02) than at baseline (Table 3, S6 Table).

**Table 3:** Changes in the primary outcomes.

Outcome Measure	Usual care group (n = 50)	Exercise group (n = 59)	Nutritional group (n = 54)
**BMI z-score**			
Baseline	2.27±0.51	2.39±0.52	2.27±0.48
6-month follow-up	2.25±0.55	2.25±0.56	2.19±0.55
Difference (6-month–baseline)	-0.018±0.17	-0.14±0.21	-0.075±0.23
p-value^b^	0.46	<0.001	0.020
**%BMI**_**p95th**_^a^			
Baseline	115±1.13	118±1.13	115±1.12
6-month follow-up	114±1.13	114±1.13	113±1.14
Difference (6-month–baseline)	-1.00±1.04	-1.03±1.05	-1.02±1.05
p-value^b^	0.49	<0.001	0.021

Abbreviations: BMI, body mass index; %BMI_p95th_, percentage of the 95th percentile of age- and sex-specific body mass index.

Data are expressed as mean±standard deviation unless otherwise indicated.

^a^Geometric mean±standard deviation.

^b^Paired t-test between baseline data and 6-month follow-up data.

### Within-group changes in the secondary outcomes: Body composition, cardiometabolic risk markers, nutrition, cardiorespiratory fitness, and muscular strength

After 6 months of intervention, all groups had lower total energy intake and post-exercise HR, as well as higher LM, adiponectin, and leg extension 1-RM (Table 4, S7 Table). In the usual care group, BMI (28.6±4.12 kg/m2 vs. 28.2±4.09 kg/m2, p = 0.044), WC (88.9±10.4 cm vs. 87.2±11.9 cm, p = 0.048), BF (28.9±8.55 kg vs. 27.9±8.58 kg, p = 0.008), and chest press 1-RM (34.4±15.1 kg vs. 28.6±10.8 kg, p = 0.001) were higher; however, LDL-C (109±19.2 mg/dL vs. 115±22.8 mg/dL, p = 0.019) was lower at the 6-month follow-up than at baseline. In the exercise group, BMI (28.1±4.23 kg/m2 vs. 28.5±4.35 kg/m2, p = 0.029), %BF (41.3±4.61% vs. 41.8±4.67%, p < 0.049), SBP (1141±9.86 mmHg vs. 119±13.3 mmHg, p = 0.002), and HDL-C (49.5±12.0 mg/dL vs. 51.4±12.5 mg/dL, p = 0.030) were lower at the 6-month follow-up than at baseline, while TG (114±1.55 mg/dL vs. 102±1.51 mg/dL, p = 0.018) was higher. In the nutritional group, TG (93.2±1.52 mg/dL vs. 104±1.64 mg/dL, p = 0.029) and ALT (25.2±1.96 U/L vs. 29.5±2.29 U/L, p = 0.025) were lower at 6-month follow-up than at baseline (Table 4, S7 Table).

**Table 4:** Changes in secondary outcomes.

Outcome Measure	Usual care group (n = 50)	Exercise group (n = 59)	Nutritional group (n = 54)
**Body mass index, kg/m**^**2**^			
Baseline	28.2±4.09	28.5±4.35	28.6±4.32
6-month follow-up	28.6±4.12	28.1±4.23	28.6±4.62
Difference (6-month–baseline)	0.39	-0.38	0.045
p-value^b^	0.044	0.029	0.82
**Body composition**			
**Waist circumference, cm**			
Baseline	87.2±11.9	86.9±10.7	89.9±10.4
6-month follow-up	88.9±10.4	86.7±10.5	88.9±10.7
Difference (6-month–baseline)	1.66±5.80	-0.22±5.36	-1.04±6.25
p-value^b^	0.048	0.76	0.23
**Body fat, kg**			
Baseline	27.9±8.58	27.1±7.96	28.5±8.99
6-month follow-up	28.9±8.55	27.5±8.02	29.0±9.45
Difference (6-month–baseline)	1.03±2.61	0.44±2.25	0.59±3.29
p-value^b^	0.008	0.14	0.20
**Body fat, %**			
Baseline	42.1±3.92	41.8±4.67	41.8±3.70
6-month follow-up	41.5±4.06	41.3±4.61	41.1±4.19
Difference (6-month–baseline)	-0.56±2.04	-0.51±1.95	-0.63±2.41
p-value^b^	0.058	0.049	0.058
**Lean mass, kg**			
Baseline	36.5±10.8	35.7±9.33	37.5±9.67
6-month follow-up	38.7±10.9	36.9±9.44	39.4±10.4
Difference (6-month–baseline)	2.27±1.75	1.26±1.62	1.88±1.72
p-value^b^	<0.001	<0.001	<0.001
**Cardiometabolic risk marker**			
**SBP, mmHg**			
Baseline	119±12.2	119±13.3	120±15.9
6-month follow-up	118±14.0	114±9.86	119±14.4
Difference (6-month–baseline)	-1.36±12.7	-4.88±11.3	-1.26±13.1
p-value^b^	0.45	0.002	0.48
**DBP, mmHg**			
Baseline	68.8±8.74	67.8±10.3	69.5±9.61
6-month follow-up	69.4±8.60	67.9±9.88	69.2±10.0
Difference (6-month–baseline)	0.62±10.8	0.10±11.3	-0.24±11.0
p-value^b^	0.69	0.95	0.87
**HOMA-IR (n = 49 / 59 / 54)**^a^			
Baseline	3.88±1.67	4.06±1.80	3.98±1.50
6-month follow-up	4.10±1.69	4.15±1.71	4.09±1.67
Difference (6-month–baseline)	1.06±1.56	1.02±1.75	1.03±1.54
p-value^b^	0.39	0.76	0.66
**TC, mg/dL (n = 32 / 28 / 36)**			
Baseline	179±26.8	178±25.2	170±28.6
6-month follow-up	177±17.9	178±23.2	173±27.5
Difference (6-month–baseline)	-1.44±20.1	-0.36±17.8	2.42±13.7
p-value^b^	0.69	0.92	0.30
**HDL-C, mg/dL (n = 49 / 59 / 54)**			
Baseline	50.2±10.5	51.4±12.5	48.7±9.82
6-month follow-up	50.8±11.3	49.5±12.0	50.1±8.73
Difference (6-month–baseline)	0.55±7.14	-1.86±6.44	1.46±5.56
p-value^b^	0.59	0.030	0.058
**LDL-C, mg/dL (n = 49 / 59 / 54)**			
Baseline	115±22.8	112±23.2	110±25.9
6-month follow-up	109±19.2	109±24.5	108±24.6
Difference (6-month–baseline)	-5.71±16.5	-3.29±17.6	-1.93±13.2
p-value^b^	0.019	0.16	0.29
**TG, mg/dL (n = 49 / 59 / 54)**^a^			
Baseline	98.2±1.62	102±1.51	104±1.64
6-month follow-up	105±1.60	114±1.55	93.2±1.52
Difference (6-month–baseline)	1.07±1.59	1.12±1.43	-1.12±1.45
p-value^b^	0.30	0.018	0.029
**AST, U/L (n = 49 / 59 / 54)**^a^			
Baseline	23.0±1.57	23.42±1.57	26.1±1.61
6-month follow-up	21.5±1.27	22.34±1.52	23.8±1.47
Difference (6-month–baseline)	-1.07±1.40	-1.05±1.32	-1.09±1.43
p-value^b^	0.17	0.20	0.070
**ALT, U/L (n = 49 / 59 / 54)**^a^			
Baseline	22.6±1.96	23.9±2.17	29.5±2.29
6-month follow-up	20.8±1.58	21.6±1.99	25.2±1.96
Difference (6-month–baseline)	-1.08±1.59	-1.10±1.58	-1.17±1.66
p-value^b^	0.23	0.10	0.025
**GGT, U/L (n = 49 / 59 / 54)**^a^			
Baseline	19.6±1.51	20.1±1.52	23.6±1.72
6-month follow-up	19.3±1.35	19.5±1.58	22.6±1.58
Difference (6-month–baseline)	-1.01±1.28	-1.03±1.33	-1.04±1.38
p-value^b^	0.71	0.39	0.33
**CRP, mg/L (n = 49 / 59 / 54)**^a^			
Baseline	1.39±2.08	1.42±2.40	1.79±2.18
6-month follow-up	1.46±2.99	1.48±2.46	1.43±2.42
Difference (6-month–baseline)	1.05±2.43	1.04±1.99	-1.25±2.25
p-value^b^	0.71	0.66	0.051
**Adiponectin, μg/mL (n = 48 / 58 / 54)**^a^			
Baseline	8.22±1.46	6.97±1.51	8.15±1.55
6-month follow-up	9.44±1.47	10.11±1.56	9.47±1.49
Difference (6-month–baseline)	1.15±1.57	1.45±1.65	1.16±1.66
p-value^b^	0.038	<0.001	0.035
**Nutrition**			
**Total energy intake, kcal (n = 50 / 59 / 53)**^a^			
Baseline	2112±1.30	2089±1.26	2205±1.23
6-month follow-up	1665±1.38	1659±1.29	1730±1.34
Difference (6-month–baseline)	-1.27±1.41	-1.26±1.33	-1.27±1.32
p-value^b^	<0.001	<0.001	<0.001
**Cardiorespiratory fitness and muscular strength**			
**Step test, post exam HR, BPM (n = 48 / 58 / 53)**			
Baseline	113±17.6	117±16.3	112±16.0
6-month follow-up	106±16.7	108±14.3	103±14.2
Difference (6-month–baseline)	-7.00±14.1	-9.07±15.8	-9.02±12.4
p-value^b^	0.001	<0.001	<0.001
**Chest press, 1-RM, kg (n = 49 / 59 / 54)**			
Baseline	28.6±10.8	29.4±11.7	29.5±11.3
6-month follow-up	34.4±15.1	28.2±12.9	29.4±14.2
Difference (6-month–baseline)	5.77±11.6	-1.19±8.73	-0.19±8.85
p-value^b^	0.001	0.30	0.88
**Leg extension, 1-RM, kg (n = 49 / 58 / 54)**			
Baseline	42.4±17.5	44.1±19.4	44.8±18.4
6-month follow-up	57.1±18.5	52.6±19.6	56.5±21.4
Difference (6-month–baseline)	14.7±15.7	8.45±12.0	11.7±14.6
p-value^b^	<0.001	<0.001	<0.001

Abbreviations: SBP, systolic blood pressure; DBP, diastolic blood pressure; HOMA-IR, homeostasis model assessment for insulin resistance; TC, total cholesterol; HDL-C, high-density lipoprotein cholesterol; LDL-C, low-density lipoprotein cholesterol; TG, triglyceride; AST, aspartate aminotransferase; ALT, alanine aminotransferase; GGT, gamma-glutamyl transferase; CRP, high-sensitivity C-reactive protein; HR, heart rate; BPM, beats per minute; RM, repetition maximum.

HOMA-IR = (Fasting Plasma Glucose Level (mg/dL) × Fasting Plasma Insulin Level (μU/mL)) / 405.

Data are expressed as mean±standard deviation unless otherwise indicated.

^a^Geometric mean±standard deviation.

^b^Paired t-test between baseline data and 6-month follow-up data.

### Between-group differences in outcomes over time

Mixed effects linear regression models for repeated measures were used to analyze the between-group differences in outcomes over time. After adjustment for confounding variables including age, sex, parental obesity, parental education, monthly household income, living with both parents, and sleep time, significant group by time interaction effects were observed in primary and secondary outcomes. The decrease in the primary outcome, BMI z-score, was more significant in the exercise group than in the usual care group over the 6-month period (β, -0.11; 95% CI, -0.20 to -0.023). Likewise, the decrease in %BMI_p95th_ was also more significant in the exercise group than in the usual care group over the 6-month period (β, -1.03; 95% CI, -1.05 to -1.01). Increases in the secondary outcome, adiponectin, was more significant in the exercise group compared to the usual care group over the 6-month period (β, 1.31; 95% CI, 1.08 to 1.58). The decrease in WC was more significant in the nutritional group compared to the usual care group over the 6-month period (β, -3.47; 95% CI, -6.06 to -0.89) (Table 5). The same results were obtained when using the ITT approach (S8 Table).

**Table 5:** Between-group differences in outcomes over time.

Outcome Measure	Model 1		Model 2		Model 3	
	β (95% CI)	p-value	β (95% CI)	p-value	β (95% CI)	p-value
**BMI z-score**						
Usual care group	reference		reference		reference	
Exercise group	-0.12 (-0.20 to -0.047)	0.002	-0.11 (-0.20 to -0.023)	0.013	-0.11 (-0.18 to -0.038)	0.003
Nutritional group	-0.057 (-0.13 to 0.021)	0.15	-0.060 (-0.15 to 0.030)	0.19	-0.056 (-0.13 to 0.016)	0.129
**%BMI**_**p95th**_						
Usual care group	reference		reference		reference	
Exercise group	-1.03 (-1.05 to -1.01)	0.001	-1.03 (-1.05 to -1.01)	0.007	-1.03 (-1.04 to -1.01)	0.001
Nutritional group	-1.01 (-1.03 to 1.01)	0.17	-1.01 (-1.03 to 1.01)	0.19	-1.01 (-1.03 to 1.00)	0.13
**Waist circumference, cm**						
Usual care group	reference		reference		reference	
Exercise group	-1.90 (-4.03 to 0.23)	0.080	-1.30 (-3.86 to 1.25)	0.32	-1.73 (-3.68 to 0.22)	0.083
Nutritional group	-2.37 (-4.54 to -0.21)	0.032	-3.47 (-6.06 to -0.89)	0.009	-3.71 (-5.68 to -1.74)	<0.001
**Adiponectin, μg/mL**						
Usual care group	reference		reference		reference	
Exercise group	1.28 (1.08 to 1.51)	0.005	1.31 (1.08 to 1.58)	0.005	1.27 (1.10 to 1.47)	0.001
Nutritional group	-1.00 (-1.19 to 1.18)	0.97	-1.08 (-1.30 to 1.12)	0.44	-1.08 (-1.25 to 1.07)	0.30

Abbreviations: BMI, body mass index; %BMI_p95th_, percentage of the 95th percentile of age- and sex-specific body mass index.

Model 1: group × time interaction effects adjusted for age and sex in the mixed effects linear regression models (random intercept: individual).

Model 2: group × time interaction effects adjusted for age, sex, parental obesity, parental education, monthly household income, living with both parents, and sleep time in the mixed effects linear regression models (random intercept: individual).

Model 3: group × time interaction effects adjusted for age, sex, parental obesity, parental education, monthly household income, living with both parents, sleep time, and baseline values in the mixed effects linear regression models (random intercept: individual).

## Discussion

### Findings

This 6-month follow-up assessment of a 24 month multidisciplinary lifestyle intervention program for children and adolescents with moderate to severe obesity revealed an improved obesity status and cardiometabolic risk. A greater effect was observed in the groups with strengthened intervention factors (i.e., additional exercise and nutrition) than in the usual care group.

In this study, circuit training was applied as an exercise intervention method. According to a recent meta-analysis, circuit exercise is known to effectively reduce body weight and BMI in people with obesity [30]. In a 16-week pilot intervention program by our research group, we found that BMI z-score, the primary outcome of the intervention, was significantly decreased in the exercise group with circuit training compared to the baseline after the intervention, but the changes in BMI z-score were not significantly different between groups [28]. In contrast to the pilot program, the 6-month intervention program showed significant group by time interaction effects between the exercise versus usual care groups in BMI z-score at 6-month follow-up. The American Academy of Pediatrics Council on Sports Medicine and Fitness recommends that strength training be performed 2 to 3 times per week and be at least 8 weeks in duration [31]. A recent systematic review showed that lifestyle-based weight loss interventions in children and adolescents had a positive effect on obesity only when they had 26 or more hours of intervention contact [32]. Given the recommendations and results from previous studies, 16 weeks may be too short to show a difference between groups. In a previous RCT of adolescents with overweight, there were no significant intervention effects on insulin sensitivity and body composition between the exercise group as compared to the control after 16 weeks of intervention [33]. With an increased period of exercise and compensation for the relatively low frequency of exercise, a meaningful difference seemed to appear between groups. The exercise group had more visits and more hours of intervention contact than the other groups, which may have had a positive effect in this study.

Boys aged 6 to 17 years and aged 11 years in the 95th percentile of age- and sex-specific BMI according to the 2007 Korean National Growth Charts [15] had an average change in BMI of 0.37 and 0.52 over 6 months, respectively. Girls aged 6 to 17 years and aged 11 years in the 95th percentile of age- and sex-specific BMI according to the 2007 Korean National Growth Charts [15] had an average change in BMI of 0.32 and 0.46 over 6 months, respectively. In our study, the usual care group had a change in BMI of 0.39±1.34 (mean±SD) over 6 months. However, the nutritional group had a change in BMI of 0.045±1.43 over 6 months. Moreover, the exercise group had a change in BMI of -0.38±1.29 over 6 months.

Concentration of adiponectin is known to be negatively correlated with BMI [34], and weight loss increases plasma adiponectin concentrations [35]. A recent meta-analysis has reported that physical exercise increases adiponectin concentration [36]. After 6 months of intervention in the present study, adiponectin was elevated in all groups, and increases in adiponectin level were significantly higher in the exercise group than in the usual care group. A recent review reported that low adiponectin levels in childhood and adolescence are associated with the development of obesity-related cardiovascular complications in adulthood, and the development of metabolic syndrome, insulin resistance, and hypertension in pediatric age populations [37]. Therefore, it seems that an increase in adiponectin will have a positive effect on cardiovascular outcomes, given the inverse correlation. However, longitudinal studies are needed, designed to demonstrate that increased adiponectin levels in childhood and adolescence are associated with better cardiovascular outcomes in adulthood.

Our study also showed a more significant decrease in WC in the nutritional group than in the usual care group. However, previous meta-analyses reported that nutritional interventions have no significant effect on WC [38–40]. A recent study found that a diet quality score was inversely associated with WC in men [41]. Therefore, WC appears to decrease relatively, due to increased diet quality as a result of personalized and balanced diet in the nutritional group. However, it is also possible that there was a statistically significant difference in WC change because the baseline WC of the nutritional group was relatively large compared to other groups although it was not statistically significant.

Previous systematic reviews and meta-analyses have reported that exercise could improve cardiorespiratory fitness, LM, and muscular strength [42–44]. However, in our study, the cardiorespiratory fitness, LM, and muscular strength of the lower extremities improved after 6 months of intervention in all groups. In the exercise group, the physical fitness improvement effect was not greater than that found in the other groups. This is because the usual care group was an active comparator, having received medical, exercise, and nutritional counseling, unlike the control group in other studies. We confirmed that the usual care protocol of this program was sufficient for improving physical fitness over the 6 months. In children and adolescents, as age increases, body weight and LM increase, and %BF shows various patterns according to age group and sex [45, 46]. In this study, it is meaningful that the participants in the exercise group had a decrease in %BF as well as an increase in LM even though the BMI z-score showed the greatest decrease. Moreover, previous studies have shown a significant association between cardiorespiratory fitness and muscular strength with cardiometabolic risk or insulin sensitivity in children [47, 48]. Thus, children can derive protective health benefits against cardiometabolic risks by participating in the intervention program described in the present study. Therefore, longitudinal studies are needed to demonstrate whether increased duration or intensity of circuit training in the exercise group is associated with better physical fitness and/or cardiometabolic status as compare to the other intervention groups.

In this study, a personalized nutritional counseling for balanced diets without energy restriction was conducted. Although daily total energy intake was reduced in all groups after 6 months of intervention, changes in energy intake did not show a significant difference between the three groups. Similar to the results of our study, the previous studies which did not focus on calorie restriction reported reduced energy intake after the lifestyle intervention [49, 50]. Moreover, a previous meta-analysis reported that structured physical activity interventions were more likely to lead to decreased daily energy intake in adolescents with obesity [51]. In this study, decreased consumption of high-calorie snacks as a result of nutritional counseling may have resulted in reduction in total energy intake. Moreover, it should not be overlooked that the participants who have learned the relation between excessive calorie intake and obesity may underreport their dietary intake.

Although overall obesity status was improved through the intervention of this study, there was no significant improvement in HOMA-IR. Previous studies reported positive effects of circuit training-based exercise intervention on insulin resistance in adolescents [11, 12]. However, a recent systematic review reported that the effects of nutritional education on insulin resistance in children and adolescents remain inconclusive [52]. Considering that insulin resistance in children and adolescents is related to the pubertal stage, we also performed a sensitivity analysis based on baseline pubertal stage (Tanner stage 1 as prepubertal; Tanner stage 2 and above as pubertal). There were no between-group differences in insulin resistance over time when all participants were considered. However, when considering pubertal participants only, the results were significant. The decrease in HOMA-IR was more significant in the exercise group than in the usual care group over the 6-month period after adjusting for age, sex, parental obesity, monthly household income, living with both parents, and sleep time (β, -1.32; 95% CI, -1.74 to -1.01) (S9 Table). Insulin resistance increases immediately following the onset of puberty (Tanner stage 2) but returns to near prepubertal levels by the end of puberty (Tanner stage 5) [53]. However, there is evidence that insulin resistance does not resolve in adolescents who have obesity at the time of entering puberty [54]. Therefore, according to our study, improvements in obesity status through exercise during puberty may be able to restore insulin resistance.

Contrary to our expectations, a significant improvement in upper body strength determined via 1-RM of chest press was not observed in the exercise group compared to the usual care group. Such a result may suggest that our exercise regimen is not sufficient to induce substantial improvement in upper body muscular strength in children and adolescents with obesity. However, the usual care group were also asked to perform regular home-based exercise using ICAAN Exercise during the intervention, although they did not participate in any supervised group exercise sessions. In addition, it is also plausible that the improvement in muscular strength during growth is associated with neuromuscular adaptations such as motor skill coordination and/or hormonal changes [55, 56]. However, evidence to support this is still lacking in our study since we did not account for the neuromuscular adaptations to determine strength change or measure-related hormones. Further studies are necessary to address this issue.

### Directions for future research

Given the high attrition rate and the non-RCT design, the results of this study should be interpreted cautiously. Therefore, long-term RCTs of multidisciplinary intervention including exercise and nutritional counseling specialized for children and adolescents with obesity are warranted. In addition, longitudinal studies are needed to demonstrate whether changes in the duration or intensity of circuit training will have better results for physical fitness and/or cardiometabolic risk in children and adolescents with obesity.

### Implications for practice

When meeting children and adolescents with obesity in the outpatient clinic, circuit training education and balanced diet counseling for at least 6 months are expected to reduce the prevalence of obesity and cardiometabolic risk in children and adolescents. Nutritional counseling should focus on a balanced diet, not on energy restriction. In addition, circuit training should be conducted at least 2–3 times a week.

### Implications for policy

It is expected to reduce the prevalence of obesity and cardiometabolic risk in children and adolescents by allocating time in a policy manner so that group exercises and nutritional counseling sessions can be conducted regularly at school. When providing meals to students at school, the focus should be on a balanced diet rather than on energy restriction. In addition, group circuit training should be included in the physical education curriculum to be conducted at least 2–3 times a week.

### Strengths and limitations

This study had some limitations. Despite the consecutive randomization procedure, the participants could not be allocated completely randomly into the three groups for geographical reasons and personal circumstances. However, there were no significant differences in the proportion of participants with severe obesity, demographic characteristics, anthropometric measurements, laboratory test results, lifestyle measurements, or fitness test results between groups at baseline. Another limitation is the relatively short follow-up duration. However, this study provided an interim result of a long-term intervention; therefore, the long-term effect of the intervention will be evaluated. Finally, the effect of the intervention may have been overestimated due to the high attrition rate. However, there was no difference in all characteristics and outcome variables between completers and dropouts at baseline (S4 and S5 Tables). In addition, there was no difference between intervention groups in the baseline values of the main variables of all participants and dropouts (S10–S13 Tables).

The strength of this study is that the protocol can be easily applied in a real-world setting. With our exercise program, we have developed a long-term sustainable program by applying more elaborate circuit training, which differs somewhat yet similar to our previous short-term program. Therefore, this program can be performed easily by children and adolescents with moderate to severe obesity at home. As a result, our program is expected to help maintain a healthy weight and cardiometabolic health for a long time.

## Conclusions

In previous studies, machines or free weights were used during circuit training, and short-term interventions of less than 6 months were performed, but in this study, circuit training that anyone can easily follow, is effective, and uses only one’s body, was applied for a longer period. Multidisciplinary intervention including circuit training and a balanced diet for children and adolescents with obesity reduced the BMI z-score and improved selected cardiometabolic risk markers such as adiponectin and WC. Insulin resistance was also improved by improving obesity status through circuit training during puberty. Circuit training should be conducted at least 2–3 times a week and nutritional counseling should focus on a balanced diet, not on energy restriction. Exercise and nutritional counseling specialized for children and adolescents with obesity should last for at least 6 months in order to see these effects. However, considering the dropouts, there may be some limitations in generalizing the results of this study.

## Supporting information

S1 Fig Intervention flow chart. (DOCX)

S1 Table The contents of the workbook. (DOCX)

S2 Table Monthly key messages and action plan for nutritional counseling. (DOCX)

S3 Table Protocol for ICAAN exercise. (DOCX)

S4 Table Baseline demographic characteristics and anthropometric measurements of completers and dropouts. (DOCX)

S5 Table Baseline laboratory test results, lifestyle measurements, and fitness test results of completers and dropouts. (DOCX)

S6 Table Changes in the primary outcomes. (DOCX)

S7 Table Changes in secondary outcomes. (DOCX)

S8 Table Between-group differences in outcomes over time (the intention-to-treat approach). (DOCX)

S9 Table Between-group differences in homeostasis model assessment for insulin resistance over time according to baseline pubertal stage. (DOCX)

S10 Table Baseline demographic characteristics and anthropometric measurements of all the recruited participants. (DOCX)

S11 Table Baseline laboratory test results, lifestyle measurements, and fitness test results of all the recruited participants. (DOCX)

S12 Table Baseline demographic characteristics and anthropometric measurements of the participants lost to follow-up. (DOCX)

S13 Table Baseline laboratory test results, lifestyle measurements, and fitness test results of the participants lost to follow-up. (DOCX)

S1 Checklist TREND checklist. (DOCX)

S1 File Study protocol (Korean). (DOCX)

S2 File Study protocol (English). (DOCX)
