# Supplementary material for: Effects of circuit training or a nutritional intervention on body mass index and other cardiometabolic outcomes in children and adolescents with overweight or obesity
Source: PLoS One. 2021 Jan 28;16(1):e0245875. doi: 10.1371/journal.pone.0245875 (PMC7842905; doi:10.1371/journal.pone.0245875)
Supplement: S1 Fig — (DOCX) [file pone.0245875.s001.docx]

**S1 Fig.** Intervention flow chart

Month 0

Medical consultation

(Health risk assessment

& Lifestyle assessment)

***Usual care group (U)***

***Nutritional group (N)***

Screening visit

Body measurement

Laboratory test

Fitness test

Questionaires

Recruitment

Month 6

Body measurement

Laboratory test

Fitness test

Questionaires

Medical consultation

Month 12

Body measurement

Laboratory test

Fitness test

Questionaires

Medical consultation

Month 18

Body measurement

Laboratory test

Fitness test

Questionaires

Medical consultation

Month 24

Body measurement

Laboratory test

Fitness test

Questionaires

Medical consultation

***Exercise group (E)***

Month 1-6

Monthly

exercise & nutrition

counseling

Month 7-12

Monthly

group activity

Month 13-15

Monthly

exercise & nutrition

counseling

Month 16-24

Monthly

group activity

Month 1-3

(U) + Weekly

group exercise &

exercise feedback

Month 4-6

(U) + Biweekly

group exercise &

exercise feedback

Month 13-15

(U) + Biweekly

group exercise &

exercise feedback

Month 7-12

(U) + Monthly

exercise feedback

Month 16-24

(U) + Monthly

exercise feedback

Month 1-6

(U) + Monthly

intensive nutrition

counseling &

Weekly

nutrition feedback

Month 7-12

(U) + Every 3 months

intensive nutrition

counseling &

Weekly

nutrition feedback

Month 13-15

(U) + Monthly

intensive nutrition

counseling &

Weekly

nutrition feedback

Month 16-24

(U) + Weekly

nutrition feedback

End of follow-up
