# Supplementary material for: Effects of circuit training or a nutritional intervention on body mass index and other cardiometabolic outcomes in children and adolescents with overweight or obesity
Source: PLoS One. 2021 Jan 28;16(1):e0245875. doi: 10.1371/journal.pone.0245875 (PMC7842905; doi:10.1371/journal.pone.0245875)
Supplement: S1 Table — (DOCX) [file pone.0245875.s002.docx]

**S1 Table.** The contents of the workbook

| Visit | Title | Contents |
| --- | --- | --- |
| Screening | Know my body 1 | Baseline tests |
| Month 0 | Learn my body 1 | One-to-one medical consultation / Goal setting  *Health risk assessment*  *Lifestyle assessment* |
| Month 1 | Manage my body 1 | Self-check  *What makes me so hard?*  *What is the obstacle to my weight control?*  *What is the lifestyle of my family?*  *What is stressful?* |
| Month 2 | Manage my body 2 | Physical activity  *Activity check and feedback*  *Individual exercise plan* |
| Month 3 | Manage my body 3 | Motivation / Mindfulness  *Encouraging health-related behavior choices*  *Identifying problems or strengths* |
| Month 4 | Manage my body 4 | Activity / Inactivity  *Increasing activity*  *Decreasing inactivity* |
| Month 5 | Manage my body 5 | Self-observation  *What changes in my body?*  *My appearance that I think, and my appearance that others think*  *How much have I changed?* |
| Month 6 | Know my body 2 | Follow-up tests |
