# Supplementary material for: Effects of circuit training or a nutritional intervention on body mass index and other cardiometabolic outcomes in children and adolescents with overweight or obesity
Source: PLoS One. 2021 Jan 28;16(1):e0245875. doi: 10.1371/journal.pone.0245875 (PMC7842905; doi:10.1371/journal.pone.0245875)
Supplement: S2 Table — (DOCX) [file pone.0245875.s003.docx]

**S2 Table.** Monthly key messages and action plan for nutritional counseling

| Session | Usual care / Exercise group | Nutrition group |
| --- | --- | --- |
| Screening | Stages of behavior change, nutrition quotient (NQ), self-efficacy, nutrient intake status | Stages of behavior change, NQ, self-efficacy, nutrient intake status |
| Month 0 | Food diary writing and five food group intake training | Individual management according to Nutritional Care Process (NCP) → Food diary writing and five food group intake training |
| Month 1 | Checking food diary, nutrition education with the theme ‘healthy protein story’ | Individual management according to NCP → Checking food diary, nutrition education with the theme ‘healthy protein story’ |
| Month 2 | Checking food diary, nutrition education with the theme ‘five colored vegetable / fruit story’ | Individual management according to NCP → Checking food diary, nutrition education with the theme ‘five colored vegetable / fruit story’ |
| Month 3 | Checking food diary, nutrition education with the theme ‘controlling the amount of food by hand’ | Individual management according to NCP → Checking food diary, nutrition education with the theme ‘controlling the amount of food by hand’ |
| Month 4 | Checking food diary, nutrition education with the theme ‘Nutrition Facts Table’ | Individual management according to NCP → Checking food diary, nutrition education with the theme ‘Nutrition Facts Table’ |
| Month 5 | Checking food diary, nutrition education with the theme ‘determining high-calorie low-nutrition food’ | Individual management according to NCP → Checking food diary, nutrition education with the theme ‘determining high-calorie low-nutrition food’ |
| Month 6 | Stages of behavior change, NQ, self-efficacy, nutrient intake status | Individual management according to NCP → Stages of behavior change, NQ, self-efficacy, nutrient intake status |
