# Supplementary material for: Effects of circuit training or a nutritional intervention on body mass index and other cardiometabolic outcomes in children and adolescents with overweight or obesity
Source: PLoS One. 2021 Jan 28;16(1):e0245875. doi: 10.1371/journal.pone.0245875 (PMC7842905; doi:10.1371/journal.pone.0245875)
Supplement: S3 Table — (DOCX) [file pone.0245875.s004.docx]

**S3 Table.** Protocol for ICAAN exercise

| Module | ICAAN Exercise protocol |
| --- | --- |
| 1 | Butt Kicker → Wood Chop → Plank Jack → Squat → Lunge → Plank |
| 2 | Standing Bird Dog → Up-down Plank → Side Lunge → Rotational Chop → Running Mountain Climber → Plank |
| 3 | Alternating Fast Feet → In-and-Out Squat → Hands-up Side Bend → Side-to-Side Mountain Climber → Wall Sit → Plank |
| 4 | Jumping Jack → Buffy → Frogger Plank → Wall Squat → Side-Kick Mountain Climber → Plank |
| 5 | High Knee Run → Mountain Climber → Arm Walking → Jump Squat → Long Jump Buffy → Plank |
| 6 | Ski Jump → Wide Squat Pulse → Flow Push-up → Buffy Knee Kick → Scissors → Plank |

ICAAN, Intervention for Childhood and Adolescents Obesity via Activity and Nutrition
