# Supplementary material for: Effects of circuit training or a nutritional intervention on body mass index and other cardiometabolic outcomes in children and adolescents with overweight or obesity
Source: PLoS One. 2021 Jan 28;16(1):e0245875. doi: 10.1371/journal.pone.0245875 (PMC7842905; doi:10.1371/journal.pone.0245875)
Supplement: S4 Table — (DOCX) [file pone.0245875.s005.docx]

**S4 Table.** Baseline demographic characteristics and anthropometric measurements of completers and dropouts

| **Characteristic** | **Completers**  **(n = 163)** | **Dropouts**  **(n = 79)** | p-value |
| --- | --- | --- | --- |
| **Age, years** | 11.24±2.14 | 11.32±1.91 | 0.77 |
| **Age, years** |  |  | 0.62 |
| 6-9 | 53 (32.5) | 21 (26.6) |  |
| 10-14 | 101 (62.0) | 54 (68.4) |  |
| 15-17 | 9 (5.5) | 4 (5.1) |  |
| **Sex, No. (%)** |  |  | 0.85 |
| Male | 97 (59.5) | 48 (60.8) |  |
| Female | 66 (40.5) | 31 (39.2) |  |
| **Parental obesity, No. (%) (n = 145 / 63)** |  |  | 0.29 |
| None | 27 (18.6) | 8 (12.7) |  |
| Either | 118 (81.4) | 55 (87.3) |  |
| **Parental CVD history, No. (%) (n = 140 / 51)** |  |  | 0.24 |
| None | 66 (47.1) | 29 (56.9) |  |
| Either | 74 (52.9) | 22 (43.1) |  |
| **Parental education, No. (%) (n = 147 / 59)** |  |  | 0.18 |
| < College (both) | 32 (21.8) | 8 (13.6) |  |
| ≥ College (either) | 115 (78.2) | 51 (86.4) |  |
| **Monthly household income, No. (%) (n = 158 / 69)** |  |  | 0.68 |
| < 3 million KRW | 26 (16.5) | 12 (17.4) |  |
| 3-5 million KRW | 65 (41.1) | 32 (46.4) |  |
| ≥ 5 million KRW | 67 (42.4) | 25 (36.2) |  |
| **Living with both parents, No. (%) (n = 158 / 70)** |  |  | 0.97 |
| Yes | 138 (87.3) | 61 (87.1) |  |
| No | 20 (12.7) | 9 (12.9) |  |
| **Birth weight, kg (n = 154 / 63)** | 3.37±0.49 | 3.25±0.61 | 0.15 |
| **Body weight, kg** | 66.7±18.2 | 67.7±17.2 | 0.67 |
| **BMI, kg/m^2^** | 28.4±4.24 | 28.4±4.06 | 1.00 |
| **BMI z-score** | 2.31±0.51 | 2.28±0.49 | 0.67 |
| **%BMI_p95th_, %^a^** | 116.0±1.13 | 115.3±1.12 | 0.72 |
| **Waist circumference, cm** | 88.0±11.0 | 88.4±10.9 | 0.78 |
| **Body fat, kg** | 27.8±8.47 | 28.1±8.01 | 0.78 |
| **Body fat, %** | 41.9±4.12 | 41.8±4.06 | 0.95 |
| **Lean mass, kg** | 36.5±9.87 | 37.2±9.96 | 0.60 |
| **SBP, mmHg** | 119.4±13.8 | 119.3±14.5 | 0.93 |
| **DBP, mmHg** | 68.7±9.57 | 69.4±8.99 | 0.57 |

Abbreviations: CVD, cardiovascular disease; KRW, Korean Republic Won; BMI, body mass index; %BMI_p95th_, percentage of the 95th percentile of age- and sex-specific body mass index; SBP, systolic blood pressure; DBP, diastolic blood pressure.

Data are presented as mean±standard deviation for continuous variables (t-test) and number (%) for categorical variables (χ^2^ test). Percentages have been rounded up and may not total to 100.

^a^Geometric mean±standard deviation
