# Supplementary material for: Effects of circuit training or a nutritional intervention on body mass index and other cardiometabolic outcomes in children and adolescents with overweight or obesity
Source: PLoS One. 2021 Jan 28;16(1):e0245875. doi: 10.1371/journal.pone.0245875 (PMC7842905; doi:10.1371/journal.pone.0245875)
Supplement: S5 Table — (DOCX) [file pone.0245875.s006.docx]

**S5 Table.** Baseline laboratory test results, lifestyle measurements, and fitness test results of completers and dropouts

| **Characteristic** | **Completers**  **(n = 163)** | **Dropouts**  **(n = 79)** | p-value |
| --- | --- | --- | --- |
| **HOMA-IR^a^** | 3.95±1.67 | 4.06±1.83 | 0.70 |
|  |  |  |  |
| **TC, mg/dL** | 175.3±27.0 | 174.2±25.7 | 0.80 |
| **HDL-C, mg/dL** | 50.2±11.1 | 50.7±11.8 | 0.75 |
| **LDL-C, mg/dL** | 111.9±24.0 | 108.7±25.1 | 0.34 |
| **TG, mg/dL^a^** | 101.2±1.59 | 95.1±1.61 | 0.34 |
|  |  |  |  |
| **AST, U/L^a^** | 24.1±1.58 | 24.5±1.51 | 0.79 |
| **ALT, U/L^a^** | 25.0±2.16 | 25.2±2.11 | 0.94 |
| **GGT, U/L^a^** | 21.0±1.57 | 21.9±1.67 | 0.48 |
|  |  |  |  |
| **CRP, mg/L^a^** | 1.51±2.24 | 1.36±2.40 | 0.36 |
| **Adiponectin, μg/mL^a^** | 7.74±1.51 | 8.58±1.42 | 0.059 |
|  |  |  |  |
| **Total energy intake, kcal^a^** | 2133.6±1.27 | 2077.4±1.30 | 0.43 |
|  |  |  |  |
| **Sleep time, hours (n = 151 / 64)** | 8.38±2.65 | 8.61±1.56 | 0.53 |
| **Inactivity time, hours (n = 139 / 57)^a^** | 2.47±1.91 | 2.55±1.94 | 0.78 |
| **Activity level, MET-minutes/week (n = 136 / 59)^a^** | 1983.3±3.00 | 1742.7±2.84 | 0.44 |
|  |  |  |  |
| **Step test, post exam HR, BPM (n = 160 / 78)** | 114.0±16.7 | 114.4±16.9 | 0.86 |
| **Chest press, 1-RM, kg (n = 163 / 76)** | 29.2±11.2 | 30.0±11.6 | 0.65 |
| **Leg extension, 1-RM, kg (n = 162 / 78)** | 44.1±18.7 | 43.6±18.5 | 0.84 |

Abbreviations: HOMA-IR, homeostasis model assessment for insulin resistance; TC, total cholesterol; HDL-C, high-density lipoprotein cholesterol; LDL-C, low-density lipoprotein cholesterol; TG, triglyceride; AST, aspartate aminotransferase; ALT, alanine aminotransferase; GGT, gamma-glutamyl transferase; CRP, high-sensitivity C-reactive protein; MET, metabolic equivalents (1 MET: oxygen consumption of 3.5 mL/kg/minute); HR, heart rate; BPM, beats per minute; RM, repetition maximum.

HOMA-IR = (Fasting Plasma Glucose Level (mg/dL) × Fasting Plasma Insulin Level (μU/mL)) / 405.

Data are expressed as mean±standard deviation unless otherwise indicated.

^a^Geometric mean±standard deviation
