# Supplementary material for: Effects of circuit training or a nutritional intervention on body mass index and other cardiometabolic outcomes in children and adolescents with overweight or obesity
Source: PLoS One. 2021 Jan 28;16(1):e0245875. doi: 10.1371/journal.pone.0245875 (PMC7842905; doi:10.1371/journal.pone.0245875)
Supplement: S6 Table — (DOCX) [file pone.0245875.s007.docx]

**S6 Table.** Changes in the primary outcomes

| **Outcome Measure** | **Usual care group (n = 50)** | **Exercise group**  **(n = 59)** | **Nutritional group (n = 54)** |
| --- | --- | --- | --- |
| **BMI z-score** |  |  |  |
| Baseline | 2.27 (2.13 to 2.42) | 2.39 (2.25 to 2.53) | 2.27 (2.14 to 2.40) |
| 6-month follow-up | 2.25 (2.09 to 2.41) | 2.25 (2.11 to 2.40) | 2.19 (2.04 to 2.34) |
| p-value^b^ | 0.46 | <0.001 | 0.020 |
| **%BMI_p95th_^a^** |  |  |  |
| Baseline | 115 (111 to 119) | 118 (114 to 122) | 115 (111 to 119) |
| 6-month follow-up | 114 (110 to 118) | 114 (111 to 118) | 113 (109 to 117) |
| p-value^b^ | 0.49 | <0.001 | 0.021 |

Abbreviations: BMI, body mass index; %BMI_p95th_, percentage of the 95th percentile of age- and sex-specific body mass index.

Data are expressed as means (95% confidence interval) unless otherwise indicated.

^a^Geometric mean (95% confidence interval)

^b^Paired t-test between baseline data and 6-month follow-up data
