# Supplementary material for: Effects of circuit training or a nutritional intervention on body mass index and other cardiometabolic outcomes in children and adolescents with overweight or obesity
Source: PLoS One. 2021 Jan 28;16(1):e0245875. doi: 10.1371/journal.pone.0245875 (PMC7842905; doi:10.1371/journal.pone.0245875)
Supplement: S7 Table — (DOCX) [file pone.0245875.s008.docx]

**S7 Table.** Changes in secondary outcomes

| **Outcome Measure** | **Usual care group (n = 50)** | **Exercise group (n = 59)** | **Nutritional group (n = 54)** |
| --- | --- | --- | --- |
| **Body mass index, kg/m^2^** |  |  |  |
| Baseline | 28.2 (27.0 to 29.4) | 28.5 (27.4 to 29.6) | 28.6 (27.4 to 29.7) |
| 6-month follow-up | 28.6 (27.4 to 29.8) | 28.1 (27.0 to 29.2) | 28.6 (27.3 to 29.9) |
| Difference (6-month – baseline) | 0.39 | -0.38 | 0.045 |
| p-value^b^ | 0.044 | 0.029 | 0.82 |
| **Body composition** |  |  |  |
| **Waist circumference, cm** |  |  |  |
| Baseline | 87.2 (83.9 to 90.6) | 86.9 (84.1 to 89.7) | 89.9 (87.1 to 92.8) |
| 6-month follow-up | 88.9 (85.9 to 91.9) | 86.7 (84.0 to 89.4) | 88.9 (86.0 to 91.8) |
| p-value^b^ | 0.048 | 0.76 | 0.23 |
| **Body fat, kg** |  |  |  |
| Baseline | 27.9 (25.4 to 30.3) | 27.1 (25.0 to 29.1) | 28.5 (26.0 to 30.9) |
| 6-month follow-up | 28.9 (26.5 to 31.3) | 27.5 (25.4 to 29.6) | 29.0 (26.5 to 31.6) |
| p-value^b^ | 0.008 | 0.14 | 0.20 |
| **Body fat, %** |  |  |  |
| Baseline | 42.1 (41.0 to 43.2) | 41.8 (40.6 to 43.0) | 41.8 (40.8 to 42.8) |
| 6-month follow-up | 41.5 (40.4 to 42.7) | 41.3 (40.1 to 42.5) | 41.1 (40.0 to 42.3) |
| p-value^b^ | 0.058 | 0.049 | 0.058 |
| **Lean mass, kg** |  |  |  |
| Baseline | 36.5 (33.4 to 39.5) | 35.7 (33.2 to 38.1) | 37.5 (34.8 to 40.1) |
| 6-month follow-up | 38.7 (35.6 to 41.8) | 36.9 (34.5 to 39.4) | 39.4 (36.5 to 42.2) |
| p-value^b^ | <0.001 | <0.001 | <0.001 |
| **Cardiometabolic risk marker** |  |  |  |
| **SBP, mmHg** |  |  |  |
| Baseline | 119 (116 to 123) | 119 (116 to 122) | 120 (116 to 125) |
| 6-month follow-up | 118 (114 to 122) | 114 (112 to 117) | 119 (115 to 123) |
| p-value^b^ | 0.45 | 0.002 | 0.48 |
| **DBP, mmHg** |  |  |  |
| Baseline | 68.8 (66.3 to 71.2) | 67.8 (65.1 to 70.5) | 69.5 (66.9 to 72.1) |
| 6-month follow-up | 69.4 (66.9 to 71.8) | 67.9 (65.3 to 70.5) | 69.2 (66.5 to 72.0) |
| p-value^b^ | 0.69 | 0.95 | 0.87 |
| **HOMA-IR (n = 49 / 59 / 54)^a^** |  |  |  |
| Baseline | 3.88 (3.35 to 4.49) | 4.06 (3.48 to 4.74) | 3.98 (3.56 to 4.45) |
| 6-month follow-up | 4.10 (3.52 to 4.76) | 4.15 (3.61 to 4.77) | 4.09 (3.55 to 4.70) |
| p-value^b^ | 0.39 | 0.76 | 0.66 |
| **TC, mg/dL (n = 32 / 28 / 36)** |  |  |  |
| Baseline | 179 (169 to 188) | 178 (169 to 188) | 170 (161 to 180) |
| 6-month follow-up | 177 (171 to 184) | 178 (169 to 187) | 173 (164 to 182) |
| p-value^b^ | 0.69 | 0.92 | 0.30 |
| **HDL-C, mg/dL (n = 49 / 59 / 54)** |  |  |  |
| Baseline | 50.2 (47.2 to 53.2) | 51.4 (48.1 to 54.6) | 48.7 (46.0 to 51.4) |
| 6-month follow-up | 50.8 (47.5 to 54.0) | 49.5 (46.4 to 52.6) | 50.1 (47.8 to 52.5) |
| p-value^b^ | 0.59 | 0.030 | 0.058 |
| **LDL-C, mg/dL (n = 49 / 59 / 54)** |  |  |  |
| Baseline | 115 (109 to 122) | 112 (106 to 118) | 110 (103 to 117) |
| 6-month follow-up | 109 (104 to 115) | 109 (102 to 115) | 108 (101 to 114) |
| p-value^b^ | 0.019 | 0.16 | 0.29 |
| **TG, mg/dL (n = 49 / 59 / 54)^a^** |  |  |  |
| Baseline | 98.2 (85.6 to 113) | 102 (91.5 to 113) | 104 (91.2 to 119) |
| 6-month follow-up | 105 (92.0 to 121) | 114 (102 to 128) | 93.2 (83.2 to 104) |
| p-value^b^ | 0.30 | 0.018 | 0.029 |
| **AST, U/L (n = 49 / 59 / 54)^a^** |  |  |  |
| Baseline | 23.0 (20.2 to 26.2) | 23.4 (20.8 to 26.4) | 26.1 (22.9 to 29.6) |
| 6-month follow-up | 21.5 (20.1 to 23.1) | 22.3 (20.0 to 24.9) | 23.8 (21.4 to 26.4) |
| p-value^b^ | 0.17 | 0.20 | 0.070 |
| **ALT, U/L (n = 49 / 59 / 54)^a^** |  |  |  |
| Baseline | 22.6 (18.6 to 27.4) | 23.9 (19.5 to 29.2) | 29.5 (23.5 to 37.0) |
| 6-month follow-up | 20.8 (18.3 to 23.7) | 21.6 (18.1 to 25.9) | 25.2 (20.9 to 30.2) |
| p-value^b^ | 0.23 | 0.10 | 0.025 |
| **GGT, U/L (n = 49 / 59 / 54)^a^** |  |  |  |
| Baseline | 19.6 (17.8 to 21.6) | 20.1 (18.0 to 22.4) | 23.6 (20.3 to 27.3) |
| 6-month follow-up | 19.3 (17.7 to 21.1) | 19.5 (17.3 to 21.9) | 22.6 (19.9 to 25.5) |
| p-value^b^ | 0.71 | 0.39 | 0.33 |
| **CRP, mg/L (n = 49 / 59 / 54)^a^** |  |  |  |
| Baseline | 1.39 (1.13 to 1.72) | 1.42 (1.13 to 1.79) | 1.79 (1.44 to 2.21) |
| 6-month follow-up | 1.46 (1.07 to 2.00) | 1.48 (1.17 to 1.87) | 1.43 (1.13 to 1.82) |
| p-value^b^ | 0.71 | 0.66 | 0.051 |
| **Adiponectin, μg/mL (n = 48 / 58 / 54)^a^** |  |  |  |
| Baseline | 8.22 (7.36 to 9.18) | 6.97 (6.26 to 7.77) | 8.15 (7.24 to 9.19) |
| 6-month follow-up | 9.44 (8.44 to 10.6) | 10.1 (8.99 to 11.4) | 9.47 (8.49 to 10.6) |
| p-value^b^ | 0.038 | <0.001 | 0.035 |
| **Nutrition** |  |  |  |
| **Total energy intake, kcal (n = 50 / 59 / 53)^a^** |  |  |  |
| Baseline | 2112 (1958 to 2277) | 2089 (1965 to 2220) | 2205 (2085 to 2331) |
| 6-month follow-up | 1665 (1518 to 1827) | 1659 (1551 to 1774) | 1730 (1598 to 1873) |
| p-value^b^ | <0.001 | <0.001 | <0.001 |
| **Cardiorespiratory fitness and muscular strength** |  |  |  |
| **Step test, post exam HR, BPM (n = 48 / 58 / 53)** |  |  |  |
| Baseline | 113 (108 to 118) | 117 (112 to 121) | 112 (108 to 117) |
| 6-month follow-up | 106 (101 to 111) | 108 (104 to 111) | 103 (99.4 to 107) |
| p-value^b^ | 0.001 | <0.001 | <0.001 |
| **Chest press, 1-RM, kg (n = 49 / 59 / 54)** |  |  |  |
| Baseline | 28.6 (25.5 to 31.7) | 29.4 (26.4 to 32.5) | 29.5 (26.5 to 32.6) |
| 6-month follow-up | 34.4 (30.1 to 38.7) | 28.2 (24.9 to 31.6) | 29.4 (25.5 to 33.2) |
| p-value^b^ | 0.001 | 0.30 | 0.88 |
| **Leg extension, 1-RM , kg (n = 49 / 58 / 54)** |  |  |  |
| Baseline | 42.4 (37.4 to 47.4) | 44.1 (39.0 to 49.2) | 44.8 (39.8 to 49.8) |
| 6-month follow-up | 57.1 (51.8 to 62.4) | 52.6 (47.4 to 57.7) | 56.5 (50.7 to 62.4) |
| p-value^b^ | <0.001 | <0.001 | <0.001 |

Abbreviations: SBP, systolic blood pressure; DBP, diastolic blood pressure; HOMA-IR, homeostasis model assessment for insulin resistance; TC, total cholesterol; HDL-C, high-density lipoprotein cholesterol; LDL-C, low-density lipoprotein cholesterol; TG, triglyceride; AST, aspartate aminotransferase; ALT, alanine aminotransferase; GGT, gamma-glutamyl transferase; CRP, high-sensitivity C-reactive protein; HR, heart rate; BPM, beats per minute; RM, repetition maximum.

HOMA-IR = (Fasting Plasma Glucose Level [mg/dL] × Fasting Plasma Insulin Level [μU/mL]) / 405.

Data are expressed as means (95% confidence interval) unless otherwise indicated.

^a^Geometric mean (95% confidence interval)

^b^Paired t-test between baseline data and 6-month follow-up data
