# Supplementary material for: Effects of circuit training or a nutritional intervention on body mass index and other cardiometabolic outcomes in children and adolescents with overweight or obesity
Source: PLoS One. 2021 Jan 28;16(1):e0245875. doi: 10.1371/journal.pone.0245875 (PMC7842905; doi:10.1371/journal.pone.0245875)
Supplement: S8 Table — (DOCX) [file pone.0245875.s009.docx]

**S8 Table.** Between-group differences in outcomes over time (the intention-to-treat approach)

| **Outcome Measure** | Model 1 |  | Model 2 |  | Model 3 |  |
| --- | --- | --- | --- | --- | --- | --- |
|  | β (95% CI) | p-value | β (95% CI) | p-value | β (95% CI) | p-value |
| **BMI z-score** |  |  |  |  |  |  |
| Usual care group | reference |  | reference |  | reference |  |
| Exercise group | -0.10 (-0.15 to -0.046) | <0.001 | -0.089 (-0.15 to -0.030) | 0.003 | -0.090 (-0.15 to -0.029) | 0.004 |
| Nutritional group | -0.037 (-0.089 to 0.015) | 0.16 | -0.046 (-0.11 to 0.013) | 0.13 | -0.045 (-0.11 to 0.016) | 0.15 |
| **%BMI_p95th_** |  |  |  |  |  |  |
| Usual care group | reference |  | reference |  | reference |  |
| Exercise group | -1.02 (-1.04 to -1.01) | <0.001 | -1.02 (-1.04 to -1.01) | 0.001 | -1.02 (-1.04 to -1.01) | 0.001 |
| Nutritional group | -1.01 (-1.02 to 1.00) | 0.18 | -1.01 (-1.02 to 1.00) | 0.12 | -1.01 (-1.02 to 1.00) | 0.14 |
| **Waist circumference, cm** |  |  |  |  |  |  |
| Usual care group | reference |  | reference |  | reference |  |
| Exercise group | -1.48 (-2.97 to 0.013) | 0.052 | -1.19 (-2.96 to 0.57) | 0.19 | -1.09 (-2.79 to 0.61) | 0.21 |
| Nutritional group | -1.70 (-3.14 to -0.25) | 0.021 | -2.37 (-4.14 to -0.61) | 0.008 | -2.22 (-3.92 to -0.51) | 0.011 |
| **Adiponectin, μg/mL** |  |  |  |  |  |  |
| Usual care group | reference |  | reference |  | reference |  |
| Exercise group | 1.24 (1.09 to 1.41) | 0.001 | 1.28 (1.11 to 1.49) | 0.001 | 1.27 (1.10 to 1.46) | 0.001 |
| Nutritional group | 1.02 (-1.11 to 1.15) | 0.79 | 1.02 (-1.14 to 1.19) | 0.78 | 1.02 (-1.13 to 1.18) | 0.75 |

Abbreviations: BMI, body mass index; %BMI_p95th_, percentage of the 95th percentile of age- and sex-specific body mass index.

Model 1: group × time interaction effects adjusted for age and sex in the mixed effects linear regression models (random intercept: individual).

Model 2: group × time interaction effects adjusted for age, sex, parental obesity, parental education, monthly household income, living with both parents, and sleep time in the mixed effects linear regression models (random intercept: individual).

Model 3: group × time interaction effects adjusted for age, sex, parental obesity, parental education, monthly household income, living with both parents, sleep time, and baseline values in the mixed effects linear regression models (random intercept: individual).
