# Supplementary material for: Effects of circuit training or a nutritional intervention on body mass index and other cardiometabolic outcomes in children and adolescents with overweight or obesity
Source: PLoS One. 2021 Jan 28;16(1):e0245875. doi: 10.1371/journal.pone.0245875 (PMC7842905; doi:10.1371/journal.pone.0245875)
Supplement: S9 Table — (DOCX) [file pone.0245875.s010.docx]

**S9 Table.** Between-group differences in Homeostasis Model Assessment for Insulin Resistance over time according to baseline pubertal stage

| **Outcome Measure** | Model 1 |  | Model 2 |  |
| --- | --- | --- | --- | --- |
|  | β (95% CI) | p-value | β (95% CI) | p-value |
| **Per protocol** |  |  |  |  |
| **HOMA-IR (prepubertal)** |  |  |  |  |
| Usual care group | reference |  | reference |  |
| Exercise group | 1.32 (-1.02 to 1.76) | 0.064 | 1.20 (-1.13 to 1.62) | 0.24 |
| Nutritional group | 1.01 (-1.35 to 1.36) | 0.97 | -1.05 (-1.44 to 1.31) | 0.77 |
| **HOMA-IR (pubertal)** |  |  |  |  |
| Usual care group | reference |  | reference |  |
| Exercise group | -1.40 (-1.74 to -1.12) | 0.003 | -1.32 (-1.74 to -1.01) | 0.046 |
| Nutritional group | -1.07 (-1.32 to 1.16) | 0.55 | -1.05 (-1.36 to 1.23) | 0.72 |
| **Intention-to-treat** |  |  |  |  |
| **HOMA-IR (prepubertal)** |  |  |  |  |
| Usual care group | reference |  | reference |  |
| Exercise group | 1.25 (-1.02 to 1.59) | 0.068 | 1.19 (-1.08 to 1.54) | 0.17 |
| Nutritional group | 1.05 (-1.22 to 1.33) | 0.72 | 1.02 (-1.28 to 1.32) | 0.90 |
| **HOMA-IR (pubertal)** |  |  |  |  |
| Usual care group | reference |  | reference |  |
| Exercise group | -1.19 (-1.37 to -1.04) | 0.014 | -1.24 (-1.46 to -1.05) | 0.012 |
| Nutritional group | -1.01 (-1.15 to 1.12) | 0.85 | -1.00 (-1.16 to 1.16) | 0.98 |

Abbreviations: HOMA-IR, homeostasis model assessment for insulin resistance.

HOMA-IR = (Fasting Plasma Glucose Level [mg/dL] × Fasting Plasma Insulin Level [μU/mL]) / 405.

Model 1: group × time interaction effects adjusted for age and sex in the mixed effects linear regression models (random intercept: individual).

Model 2: group × time interaction effects adjusted for age, sex, parental obesity, monthly household income, living with both parents, and sleep time in the mixed effects linear regression models (random intercept: individual).
