# Supplementary material for: Effects of circuit training or a nutritional intervention on body mass index and other cardiometabolic outcomes in children and adolescents with overweight or obesity
Source: PLoS One. 2021 Jan 28;16(1):e0245875. doi: 10.1371/journal.pone.0245875 (PMC7842905; doi:10.1371/journal.pone.0245875)
Supplement: S10 Table — (DOCX) [file pone.0245875.s011.docx]

**S10 Table.** Baseline demographic characteristics and anthropometric measurements of all the recruited participants

|  | **All participants** | | |  |
| --- | --- | --- | --- | --- |
| **Characteristic** | **Usual care group**  **(n = 84)** | **Exercise group**  **(n = 74)** | **Nutritional group**  **(n = 84)** | p-value |
| **Age, years** | 11.3±1.99 | 10.9±2.19 | 11.5±2.01 | 0.25 |
| **Age, years** |  |  |  | 0.036 |
| 6-9 | 20 (23.8) | 30 (40.5) | 24 (28.6) |  |
| 10-14 | 62 (73.8) | 41 (55.4) | 52 (61.9) |  |
| 15-17 | 2 (2.4) | 3 (4.1) | 8 (9.5) |  |
| **Sex, No. (%)** |  |  |  | 0.14 |
| Male | 51 (60.7) | 38 (51.4) | 56 (66.7) |  |
| Female | 33 (39.3) | 36 (48.7) | 28 (33.3) |  |
| **Parental obesity, No. (%) (n = 71 / 66 / 71)** |  |  |  | 0.14 |
| None | 10 (14.1) | 8 (12.1) | 17 (23.9) |  |
| Either | 61 (85.9) | 58 (87.9) | 54 (76.1) |  |
| **Parental CVD history, No. (%) (n = 61 / 63 / 67)** |  |  |  | 0.20 |
| None | 26 (42.6) | 30 (47.6) | 39 (58.2) |  |
| Either | 35 (57.4) | 33 (52.4) | 28 (41.8) |  |
| **Parental education, No. (%) (n = 71 / 69 / 66)** |  |  |  | 0.37 |
| < College (both) | 11 (15.5) | 17 (24.6) | 12 (18.2) |  |
| ≥ College (either) | 60 (84.5) | 52 (75.4) | 54 (81.8) |  |
| **Monthly household income, No. (%) (n = 75 / 74 / 78)** |  |  |  | 0.19 |
| < 3 million KRW | 8 (10.7) | 17 (23.0) | 13 (16.7) |  |
| 3-5 million KRW | 30 (40.0) | 30 (40.5) | 37 (47.4) |  |
| ≥ 5 million KRW | 37 (49.3) | 27 (36.5) | 28 (35.9) |  |
| **Living with both parents, No. (%) (n = 75 / 74 / 79)** |  |  |  | 0.74 |
| Yes | 67 (89.3) | 63 (85.1) | 69 (87.3) |  |
| No | 8 (10.7) | 11 (14.9) | 10 (12.7) |  |
| **Birth weight, kg (n = 76 / 68 / 73)** | 3.28±0.60 | 3.36±0.49 | 3.37±0.49 | 0.51 |
| **Body weight, kg** | 67.0±18.4 | 65.8±17.8 | 68.2±17.6 | 0.71 |
| **BMI, kg/m^2^** | 28.2±3.97 | 28.6±4.63 | 28.5±3.99 | 0.85 |
| **BMI z-score** | 2.25±0.50 | 2.40±0.52 | 2.27±0.48 | 0.13 |
| **%BMI_p95th_, %^a^** | 114.4±1.12 | 118.3±1.14 | 114.8±1.12 | 0.16 |
| **Waist circumference, cm** | 88.1±11.6 | 87.1±11.2 | 89.1±10.1 | 0.54 |
| **Body fat, kg** | 27.8±8.07 | 27.4±8.63 | 28.4±8.33 | 0.78 |
| **Body fat, %** | 41.9±4.17 | 41.9±4.50 | 41.8±3.65 | 0.99 |
| **Lean mass, kg** | 36.8±10.7 | 36.0±9.67 | 37.3±9.28 | 0.68 |
| **SBP, mmHg** | 119.6±12.6 | 118.5±14.1 | 120.0±15.4 | 0.80 |
| **DBP, mmHg** | 68.6±8.89 | 67.7±10.2 | 70.2±9.06 | 0.24 |

Abbreviations: CVD, cardiovascular disease; KRW, Korean Republic Won; BMI, body mass index; %BMI_p95th_, percentage of the 95th percentile of age- and sex-specific body mass index; SBP, systolic blood pressure; DBP, diastolic blood pressure.

Data are presented as mean±standard deviation for continuous variables (one-way analysis of variance test) and number (%) for categorical variables (χ^2^ test). Percentages have been rounded up and may not total to 100.

^a^Geometric mean±standard deviation
