# Supplementary material for: Effects of circuit training or a nutritional intervention on body mass index and other cardiometabolic outcomes in children and adolescents with overweight or obesity
Source: PLoS One. 2021 Jan 28;16(1):e0245875. doi: 10.1371/journal.pone.0245875 (PMC7842905; doi:10.1371/journal.pone.0245875)
Supplement: S11 Table — (DOCX) [file pone.0245875.s012.docx]

**S11 Table.** Baseline laboratory test results, lifestyle measurements, and fitness test results of all the recruited participants

|  | **All participants** | | |  |
| --- | --- | --- | --- | --- |
| **Characteristic** | **Usual care group**  **(n = 84)** | **Exercise group**  **(n = 74)** | **Nutritional group**  **(n = 84)** | p-value |
| **HOMA-IR^a^** | 3.64±1.66 | 4.21±1.87 | 4.17±1.65 | 0.16 |
|  |  |  |  |  |
| **TC, mg/dL** | 178.3±26.0 | 175.1±26.3 | 171.2±27.0 | 0.36 |
| **HDL-C, mg/dL** | 50.6±10.7 | 50.6±12.1 | 50.0±11.4 | 0.91 |
| **LDL-C, mg/dL** | 113.6±23.6 | 110.5±23.2 | 108.5±26.0 | 0.40 |
| **TG, mg/dL^a^** | 95.9±1.65 | 101.5±1.50 | 100.3±1.62 | 0.72 |
|  |  |  |  |  |
| **AST, U/L^a^** | 22.9±1.50 | 23.5±1.55 | 26.4±1.61 | 0.099 |
| **ALT, U/L^a^** | 22.0±1.93 | 24.0±2.13 | 29.9±2.30 | 0.027 |
| **GGT, U/L^a^** | 19.2±1.42 | 20.2±1.55 | 24.6±1.76 | 0.0014 |
|  |  |  |  |  |
| **CRP, mg/L^a^** | 1.26±2.07 | 1.50±2.37 | 1.66±2.42 | 0.099 |
| **Adiponectin, μg/mL^a^** | 8.41±1.44 | 7.06±1.47 | 8.52±1.52 | 0.0041 |
|  |  |  |  |  |
| **Total energy intake, kcal^a^** | 2088.6±1.30 | 2071.6±1.28 | 2181.4±1.24 | 0.35 |
|  |  |  |  |  |
| **Sleep time, hours (n = 74 / 66 / 75)** | 8.58±2.76 | 8.32±2.26 | 8.43±2.07 | 0.82 |
| **Inactivity time, hours (n = 67 / 57 / 72)^a^** | 2.41±1.86 | 2.75±1.97 | 2.38±1.93 | 0.41 |
| **Activity level, MET-minutes/week (n = 66 / 57 / 72)^a^** | 1759.4±3.36 | 1504.6±2.65 | 2477.6±2.70 | 0.025 |
|  |  |  |  |  |
| **Step test, post exam HR, BPM (n = 83 / 73 / 82)** | 112.8±18.3 | 117.0±15.8 | 112.8±15.7 | 0.20 |
| **Chest press, 1-RM, kg (n = 84 / 74 / 81)** | 29.1±11.9 | 29.1±11.3 | 30.3±10.8 | 0.74 |
| **Leg extension, 1-RM, kg (n = 84 / 73 / 83)** | 42.8±18.8 | 44.0±18.9 | 45.0±18.3 | 0.74 |

Abbreviations: HOMA-IR, homeostasis model assessment for insulin resistance; TC, total cholesterol; HDL-C, high-density lipoprotein cholesterol; LDL-C, low-density lipoprotein cholesterol; TG, triglyceride; AST, aspartate aminotransferase; ALT, alanine aminotransferase; GGT, gamma-glutamyl transferase; CRP, high-sensitivity C-reactive protein; MET, metabolic equivalents (1 MET: oxygen consumption of 3.5 mL/kg/minute); HR, heart rate; BPM, beats per minute; RM, repetition maximum.

HOMA-IR = (Fasting Plasma Glucose Level (mg/dL) × Fasting Plasma Insulin Level (μU/mL)) / 405.

Data are expressed as mean±standard deviation unless otherwise indicated.

^a^Geometric mean±standard deviation
