# Supplementary material for: Effects of circuit training or a nutritional intervention on body mass index and other cardiometabolic outcomes in children and adolescents with overweight or obesity
Source: PLoS One. 2021 Jan 28;16(1):e0245875. doi: 10.1371/journal.pone.0245875 (PMC7842905; doi:10.1371/journal.pone.0245875)
Supplement: S12 Table — (DOCX) [file pone.0245875.s013.docx]

**S12 Table.** Baseline demographic characteristics and anthropometric measurements of the participants lost to follow-up

|  | **Dropouts** | | |  |
| --- | --- | --- | --- | --- |
| **Characteristic** | **Usual care group**  **(n = 34)** | **Exercise group**  **(n = 15)** | **Nutritional group**  **(n = 30)** | p-value |
| **Age, years** | 11.5±2.00 | 10.6±1.79 | 11.5±1.84 | 0.28 |
| **Age, years** |  |  |  | 0.52 |
| 6-9 | 8 (23.5) | 6 (40.0) | 7 (23.3) |  |
| 10-14 | 25 (73.5) | 9 (60.0) | 20 (66.7) |  |
| 15-17 | 1 (2.9) | 0 (0.0) | 3 (10.0) |  |
| **Sex, No. (%)** |  |  |  | 0.80 |
| Male | 21 (61.8) | 8 (53.3) | 19 (63.3) |  |
| Female | 13 (38.2) | 7 (46.7) | 11 (36.7) |  |
| **Parental obesity, No. (%) (n = 28 / 14 / 21)** |  |  |  | 0.80 |
| None | 4 (14.3) | 1 (7.1) | 3 (14.3) |  |
| Either | 24 (85.7) | 13 (92.9) | 18 (85.7) |  |
| **Parental CVD history, No. (%) (n = 22 / 13 / 16)** |  |  |  | 0.33 |
| None | 10 (45.5) | 8 (61.5) | 11 (68.8) |  |
| Either | 12 (54.6) | 5 (38.5) | 5 (31.3) |  |
| **Parental education, No. (%) (n = 27 / 14 / 18)** |  |  |  | 0.002 |
| < College (both) | 1 (3.7) | 6 (42.9) | 1 (5.6) |  |
| ≥ College (either) | 26 (96.3) | 8 (57.1) | 17 (94.4) |  |
| **Monthly household income, No. (%) (n = 27 / 15 / 27)** |  |  |  | 0.096 |
| < 3 million KRW | 2 (7.4) | 6 (40.0) | 4 (14.8) |  |
| 3-5 million KRW | 12 (44.4) | 5 (33.3) | 15 (55.6) |  |
| ≥ 5 million KRW | 13 (48.2) | 4 (26.7) | 8 (29.6) |  |
| **Living with both parents, No. (%) (n = 27 / 15 / 28)** |  |  |  | 0.90 |
| Yes | 23 (85.2) | 13 (86.7) | 25 (89.3) |  |
| No | 4 (14.8) | 2 (13.3) | 3 (10.7) |  |
| **Birth weight, kg (n = 27 / 13 / 23)** | 3.19±0.72 | 3.13±0.65 | 3.40±0.40 | 0.36 |
| **Body weight, kg** | 67.5±17.2 | 68.2±21.8 | 67.8±15.1 | 0.99 |
| **BMI, kg/m^2^** | 28.2±3.85 | 28.9±5.76 | 28.4±3.36 | 0.89 |
| **BMI z-score** | 2.23±0.48 | 2.44±0.56 | 2.27±0.47 | 0.38 |
| **%BMI_p95th_, %^a^** | 114.1±1.12 | 119.5±1.15 | 114.5±1.11 | 0.39 |
| **Waist circumference, cm** | 89.4±11.1 | 87.9±13.5 | 87.6±9.47 | 0.78 |
| **Body fat, kg** | 27.7±7.38 | 28.9±11.1 | 28.2±7.08 | 0.89 |
| **Body fat, %** | 41.5±4.54 | 42.3±3.88 | 42.0±3.64 | 0.82 |
| **Lean mass, kg** | 37.4±10.7 | 37.1±11.2 | 37.1±8.68 | 0.99 |
| **SBP, mmHg** | 120.3±13.4 | 116.6±17.2 | 119.5±14.6 | 0.72 |
| **DBP, mmHg** | 68.4±9.24 | 67.4±10.0 | 71.5±7.99 | 0.26 |

Abbreviations: CVD, cardiovascular disease; KRW, Korean Republic Won; BMI, body mass index; %BMI_p95th_, percentage of the 95th percentile of age- and sex-specific body mass index; SBP, systolic blood pressure; DBP, diastolic blood pressure.

Data are presented as mean±standard deviation for continuous variables (one-way analysis of variance test) and number (%) for categorical variables (χ^2^ test or Fisher's exact test). Percentages have been rounded up and may not total to 100.

^a^Geometric mean±standard deviation
