# Supplementary material for: Effects of circuit training or a nutritional intervention on body mass index and other cardiometabolic outcomes in children and adolescents with overweight or obesity
Source: PLoS One. 2021 Jan 28;16(1):e0245875. doi: 10.1371/journal.pone.0245875 (PMC7842905; doi:10.1371/journal.pone.0245875)
Supplement: S13 Table — (DOCX) [file pone.0245875.s014.docx]

**S13 Table.** Baseline laboratory test results, lifestyle measurements, and fitness test results of the participants lost to follow-up

|  | **Dropouts** | | |  |
| --- | --- | --- | --- | --- |
| **Characteristic** | **Usual care group**  **(n = 34)** | **Exercise group**  **(n = 15)** | **Nutritional group**  **(n = 30)** | p-value |
| **HOMA-IR^a^** | 3.42±1.59 | 4.85±2.16 | 4.52±1.88 | 0.083 |
|  |  |  |  |  |
| **TC, mg/dL** | 178.6±25.9 | 157.0±27.3 | 172.7±24.3 | 0.22 |
| **HDL-C, mg/dL** | 50.6±10.7 | 47.9±10.4 | 52.3±13.6 | 0.50 |
| **LDL-C, mg/dL** | 112.2±24.9 | 104.9±23.5 | 106.6±26.3 | 0.55 |
| **TG, mg/dL^a^** | 94.4±1.69 | 100.2±1.50 | 93.5±1.59 | 0.89 |
|  |  |  |  |  |
| **AST, U/L^a^** | 22.8±1.40 | 24.1±1.45 | 26.9±1.64 | 0.27 |
| **ALT, U/L^a^** | 21.6±1.89 | 24.4±2.03 | 30.6±2.35 | 0.18 |
| **GGT, U/L^a^** | 19.0±1.43 | 20.5±1.66 | 26.7±1.83 | 0.022 |
|  |  |  |  |  |
| **CRP, mg/L^a^** | 1.13±2.02 | 1.88±2.21 | 1.44±2.85 | 0.15 |
| **Adiponectin, μg/mL^a^** | 8.64±1.42 | 7.29±1.32 | 9.24±1.46 | 0.11 |
|  |  |  |  |  |
| **Total energy intake, kcal^a^** | 2055.2±1.30 | 2005.7±1.36 | 2140.2±1.27 | 0.70 |
|  |  |  |  |  |
| **Sleep time, hours (n = 26 / 13 /25)** | 8.39±1.39 | 9.42±1.48 | 8.41±1.69 | 0.11 |
| **Inactivity time, hours (n = 24 / 11 / 22)^a^** | 2.69±1.92 | 3.08±1.85 | 2.18±1.99 | 0.33 |
| **Activity level, MET-minutes/week (n = 24 / 10 / 25)^a^** | 1777.1±3.13 | 955.5±2.77 | 2174.9±2.44 | 0.11 |
|  |  |  |  |  |
| **Step test, post exam HR, BPM (n = 34 / 15 / 29)** | 113.3±19.2 | 118.1±14.5 | 113.7±15.4 | 0.64 |
| **Chest press, 1-RM, kg (n = 34 / 15 / 27)** | 29.6±13.5 | 27.7±9..80 | 31.8±9.90 | 0.53 |
| **Leg extension, 1-RM, kg (n = 34 / 15 / 29)** | 42.0±19.4 | 43.5±17.1 | 45.5±18.5 | 0.75 |

Abbreviations: HOMA-IR, homeostasis model assessment for insulin resistance; TC, total cholesterol; HDL-C, high-density lipoprotein cholesterol; LDL-C, low-density lipoprotein cholesterol; TG, triglyceride; AST, aspartate aminotransferase; ALT, alanine aminotransferase; GGT, gamma-glutamyl transferase; CRP, high-sensitivity C-reactive protein; MET, metabolic equivalents (1 MET: oxygen consumption of 3.5 mL/kg/minute); HR, heart rate; BPM, beats per minute; RM, repetition maximum.

HOMA-IR = (Fasting Plasma Glucose Level (mg/dL) × Fasting Plasma Insulin Level (μU/mL)) / 405.

Data are expressed as mean±standard deviation unless otherwise indicated.

^a^Geometric mean±standard deviation
