# Supplementary material for: Effects of circuit training or a nutritional intervention on body mass index and other cardiometabolic outcomes in children and adolescents with overweight or obesity
Source: PLoS One. 2021 Jan 28;16(1):e0245875. doi: 10.1371/journal.pone.0245875 (PMC7842905; doi:10.1371/journal.pone.0245875)
Supplement: S1 File — (DOCX) [file pone.0245875.s016.docx]

**S1 File.** Study protocol (Korean)

고도비만 소아청소년 장기추적 중재 연구 (ICAAN)

주관연구기관: 한림대학교 의과대학

연구책임자: 한림대학교 성심병원 가정의학과 박경희 교수

(beloved920@naver.com)

발주부처: 질병관리본부

1. 목적 및 필요성

1-1. 연구 개발의 개요

☐ 국내 고도비만의 현황

교육부가 발표한 2015년 학교건강검사 표본조사에 따르면, 초, 중, 고 학생들의 최근 5년간 전체 고도비만율이 지속적으로 증가하고 있음. 특히 고도비만은 ‘07년에 0.8%에서 ’14년에 1.4%, ’15년에 1.6%로 증가한 것으로 보고되었음. 2015년 조사에서는 여학생에 비해서는 남학생에서 고도비만의 비율이 높았고, 초등학교, 중학교, 고등학교를 거치면서 고도비만의 유병률이 증가했음.

☐ 소아청소년기 고도비만의 특성

○ 고도비만의 합병증

해외 연구에 따르면 10-14 청소년 비만아의 75%가 성인기 비만으로 이행하였음. 또한 여러 연구에서 소아청소년기 비만은 성인이 되지 않은 소아청소년기 연령에서 이미 높은 혈압, 고인슐린혈증, 비알콜성 지방간 등의 발병을 높인다는 것이 보고된 바 있음. 특히 고도비만인 소아청소년은 과체중 혹은 비만 소아청소년에 비하여, 성인기 비만으로의 이행 및 심혈관 및 대사성 위험인자를 갖는 경우가 더 많다고 알려져 있음. 국내 소아를 대상으로 한 연구에서도, 정상체중, 과체중, 비만, 고도비만아에서 심혈관 및 대사성 위험인자가 3개 이상인 대사증후군의 유병률이 IDF 기준에서는 0.2%, 2.6%, 16.8%, 30.2%로 나타나 정상체중에서 고도 비만으로 이행할수록 대사증후군 유병률도 함께 증가하는 경향을 보임.

○ 대사질환의 합병증 위험성이 증가함에 따라 이로 인한 의료비용 지출의 증가와 향후 성인기 각종 만성질환과 이에 따른 합병증 동반 위험이 증가하는 것은 보건학적 문제임. 하지만 고도비만 소아청소년의 경우, 학업일정 뿐 아니라 낮은 사회경제적 수준의 가정에 속하는 경우가 많아 병원 치료의 접근성이 떨어짐. 만성질환의 예방․관리를 위해서는 고도비만 원인 및 관리지표 개발에 대한 연구 인프라가 필수적임.

○ 사회경제적 수준과의 연관성

소아청소년 비만은 가정의 사회경제적 수준과 연관이 있음. 2011년 서울지역의 지역아동센터와 청소년 방과 후 아카데미 이용 아동 545명을 분석한 결과, 부모의 교육수준이 낮은 저소득층 아동에서 과체중, 비만아 비율이 높았음. 또한, 1998년과 2007-2008년 국민건강영양조사 자료를 비교분석한 연구에서, 소득수준이 하위 4분위에 속하는 가구에서는 비만 아동 비율이 증가하였으나, 상위 4분위에 속하는 가구에서는 비율이 감소하는 것으로 파악됨. 고도비만 소아청소년과 사회경제적 수준에 대한 연구는 많지 않으나, 미국국민건강영양조사 자료에 의하면, 고도비만 유병률이 낮은 사회경제적 수준을 가진 가정에서 더 높은 것으로 보고됨.

○ 지역별 비만율을 비교하여 볼 때, 농어촌(읍·면)지역 학생들의 비만율이 도시지역 학생들의 비만율보다 높으며, 중학교로 올라가면서 비만율의 증가도 큼

2. 연구목표

○ 한국인 소아청소년에 적합한 고도비만의 중재법 적용 및 효과판정

○ 장기 추적조사를 통한 국내 고도비만 소아청소년의 건강정보 수집

○ 국민건강 실천을 위한 조기인식 교육 프로그램 개발 및 제공

3. 연구 내용

1) 연구 대상 선정 및 모집

- 사전에 연구의 취지와 방법을 상세히 설명한 후, 연구에 대한 동의가 있는 대상자에 한해서 실시함.

- 연령 : 초등학교 4학년 – 중학교 2학년 연령 (만 10세- 14세)

- 대상자 : 가) 고도비만 청소년 300여명

• 고도비만자의 정의

- 엄격한 기준 : 성별연령별 체질량지수 95백분위수의 120% 이상.

- 허용기준 : 엄격한 기준으로 모집이 용이하지 않으므로 성별-연령별 체질량지수 97백분위수 이상을 모집과 중재의 대상으로 정의함.

• 주관연구기관 주변 지역의 고도비만 중재프로그램 대상자 인원 추정

- 2015년 교육부의 비만실태 표본조사 결과를 참조하여, 평균 약 1% 정도를 고도비만으로 추정한 후, 인근지역 교육청 홈페이지에서 4학년에서 중학교 2학년에 해당하는 인원을 산출하고 그 인원의 약 1%를 추정함.

도청, 교육청, 주관연구기관 MOU를 통해 긴밀한 업무협조를 진행할 예정

교육청 : 관할 지역 초중등학교에 공문발송, 참여희망자 모집 홍보 (학교 내 낙인화의 문제 고려하여, 보건교사가 참여대상 기준에 해당하는 아이의 학부모나 아이와 충분히 상의하여 희망자에 한 해서만 아이캔 연락처를 알려주기로 함. 사전연구 시 저사 결과, 대개 학교마다 고도비만아를 보건교사가 따로 관리하고 있음 )

• 도청 : 보건소나 주민센터 등에 모집 게시물을 붙이거나 반상회 등의 지역주민모임을 통해 공지

• 주관연구기관 : 원 내 홍보물 게시를 통해 대상자 모집

- 기타 보도자료 및 각종 SNS 홍보 등 활용

- 자문회의에서 도출되는 대상자 모집방법 논의 후 이를 적극 반영할 예정

2) 다양한 유형의 중재 프로토콜 개발 및 적용

□ 연구개발 내용 : 1차 년도

○ 다학제적 자문단 구성

- 근거중심의 장기적 고도비만 중재 프로그램 기획을 위한 전문가 자문

- 실제 현장 상황 적용 가능, 높은 접근성, 좋은 효과를 지닌 프로그램 기획을 위한 자문

- 비만, 소아청소년과 연관이 있는 각 조직에서 해당 업무를 총괄하는 대표성 있는 인물을 섭외하여 자문

- 실제 프로그램에 참여하는 현장의 소리를 듣기 위한 참여 아동 및 학부모 자문단 구성 및 운영

- 중재연구 참여율 및 순응도 향상 위한 방안으로 지역사회 네트워크 자원확보

• 대상자 확보

- 지역사회 네트워크 활용

: 교육청, 지역사회 보건교사회, 교원관련 단체, 보건소 등에 협조요청 공문을 발송하고 보건교사가 해당 아동 혹은 부모에게 프로그램 홍보하고 참여 독려

- 각종 보도매체 활용, SNS 활용 등

○ 참여 대상자에 대한 프로그램에 대한 사전 설명

고도비만 프로그램 참여 의사를 밝힌 대상자의 부모에게 전화를 통해 사전에 연구의 취지와 방법을 미리 설명한 후 참여의사를 확인

○ 중재프로그램의 개발 및 단기 적용

- 구성된 자문단과 프로그램 내용을 논의하고 최종 확정

- 중재 프로그램 확정 후 주관연구기관 윤리심의 위원회의 심의를 받은 후 프로그램 진행

- 중재의 기본 구성요소

: 중재에 포함 될 기본 구성 요소를 선정하고, 이들을 조합한 내용으로 중재유형을 구분.

- 중재 방법에 따른 중재 유형의 구분 및 중재 실행 (Phase 1)

신체적, 영양학적, 체력 평가 후 아래의 세 가지 유형의 중재군으로 할당

• 표준중재군 (S) : 최대 90명

: 의사면담, 영양상담, 행동교정 피드백, 중재 순응에 대한 전화

• 표준중재 + 강화 피드백군 (I) : 최대 90명

: 표준중재 내용 ＋ 정기적 영양 및 활동 모니터, 전화 & 문자 피드백

• 표준중재+ 강화피드백+ 운동중재군 (E) : 최대 90명

: I군 중재내용 + 정기적 방문 운동

- 중재군의 할당

• 중재군은 무작위 배정하여 평행시험으로 진행하며, 두 번째 방문 시(결과상담), 모든 대상자에게 블록화 무작위 배정법의 할당에 의하여 각 군으로 배정한다.

무작위배정표는 SAS^®^ system의 Randomization program으로 발생된 난수(A, B의 random number)의 순열을 시험대상자 번호 1번부터 순차적으로 적용시킨 것으로 SAS^®^를 통해 시험 전에 미리 고안하여 생성한다.

- 중재 장소에 따른 중재군 할당

• 환경중재군 (EM): 최대 30명 (고도비만)

: 지역아동센터 등 방과 후 대부분의 시간을 집 밖에서 보내거나 맞벌이 혹은 다른 이유로 인해 부모가 함께 방문하기 힘든 고도비만 중재 대상을 위해 연구진이 해당 아이들이 주로 생활하는 곳을 정기적으로 방문하여 중재를 시행.

: 고도비만인 소아청소년만 대상으로 하는 것이 아닌 센터 내 아이들 모두를 대상으로 건강한 생활습관에 대한 교육과 집단활동을 진행하고 센터 직원 교육 등을 통해 환경 개선을 유도. 부모가 챙겨줄 수 없는 참여자임을 고려해서 본인이 스스로를 챙길 수 있는 자조능을 키우기 위한 교육을 시행.

○ 평가 지표 선정

내부 연구진과 분야별 전문가 자문단을 통해 아래에 명시된 지표 이외에 필요한 지표들을 추가 산정할 예정임.

단면적 상관관계 분석을 위한 지표

- 비만 및 신체상태

: 키, 체중, 허리둘레, DXA로 측정한 체성분값, BMI Z-score, 체력 (VO_2_max, 근력) 등

- 대사적, 심혈관질환 위험관련 지표

: 혈압, 혈중 지질농도, 공복 인슐린, 공복혈당, 기타 대사질환 관련된 biomarker들 (FGF21, leptin 등), 경동맥 내중막두께, 피부변화 (흑색극세포증 정도 등), 대사적지표를 위한 대변채취

- 생활습관 관련

: 3일치 식사기록을 통한 영양섭취 상태, 식습관 설문

: GPAQ, 신체활동 정도 설문

: 수면시간, 스크린타임(TV/컴퓨터)

- 심리 및 정신건강 관련

: 행동척도평가, 집중력 평가 등

경시적 상관관계 분석 및 중재 효과판정을 위한 지표

- 비만상태 및 신체 개선 지표 (1차적 지표)

: 체질량지수 백분위수, BMI Z-score, 허리둘레 백분위수, 체성분 (체지방률, 제지방량)

- 생화학적 상태 개선 판정 지표

: 혈압, 혈중 지질농도, 인슐린 저항성 (HOMA-IR)

- 생활습관 및 환경관련 개선표과 지표

: 수면시간, TV/컴퓨터 사용시간, 식습관, 영양섭취 상태, Nutrition Care Process 결과 평가, 중재순응도, 신체활동지표 등

○ 평가 지표 및 설문의 시행

- 자기기입식 설문의 활용: 고도비만 청소년, 부모 기입용

* 청소년 대상 : 식습관, 신체활동, 수면시간, 음주, 흡연, TV/컴퓨터 사용시간, 여가시간 활용, 정신건강(우울, 스트레스 등) 등에 대한 항목을 시행하고 본인의 체형에 대한 인식도, 과거 체중조절 시도 등에 대한 설문항목은 국민건강영양조사와 청소년 온라인조사에 포함된 설문항목을 사용할 예정임

* 부모대상 : 자녀의 과거력(월경력 포함), 가족력, 출생관련 항목 (출생체중, 재태기간, 출산형태, 수유형태 등)을 조사하고, 부모의 신체측정치 (검진일 방문하는 부모의 경우에 한해서는 직접측정), 신체활동, 식습관 관련된 생활습관과 어머니의 출산전후 변수 (출산전 체중 및 임신 시 체중증가, 임신성 당뇨병 여부 등), 부모의 과거력 등에 대한 설문을 시행할 예정임. 설문항목 중 국민건강영양조사와 겹치는 부분은 해당 연구에서 사용한 설문항목을 사용할 예정임.

- 식습관 및 영양섭취 실태 파악

: 식습관, 식행동에 대한 설문조사와 3일간의 식사일기 (주중 2일, 주말 1일 포함)를 기록하게 함. 3일분의 영양섭취는 전문 인력에 의해 입력될 예정이며 CAN-pro (한국 영양학회)를 이용하여 영양소 섭취량으로 환산함.

- 정신건강 평가를 위한 설문

: 자가설문으로 아동청소년 행동평가척도 부모용 (K-CBCL)과 청소년용 (K-YSR)을 시행하고 결과를 웹사이트(http://www.aseba.or.kr/)에 입력하여 나온 결과지를 각자 배포하고 그 결과에 대한 사후 관리 시행

: 임상심리사에 의한 집중력 등 각종 심리평가 시행

○ 신체 측정 및 검체 수집

: 대사적 합병증 동반 여부를 확인하고 향후 유전자 검사를 위한 검체 준비를 위함임.

- 신체측정 : 키, 체중, 복부둘레

- 혈압 : 자동혈압계로 2회 측정

- 혈액검사 : 측정을 위해 방문할 때 부모와 참여예정 청소년에게 연구의 취지와 방법을 구체적으로 재차 설명하고 혈액검사 인체유래물 동의서를 작성하게 할 예정임.

채혈은 12시간 공복 후 상완정맥에서 1회용 주사기를 이용하여 채혈한 후 시험관에 혈액을 수집, 유전자 검사를 위한 검체를 보관할 예정임

- 검체 보관 : 연구대상자의 혈액은 감염성 질환이 없는 고도비만 청소년에게서 채혈한 것이며 연구에 이용되는 것은 혈액에서 분리된 DNA이므로 전염성이 없어 안전상의 우려는 없음. 검체이동 및 검사결과의 정도관리를 위해 혈액검사는 실험실 안전에 관한 지침을 잘 숙지하고 기존에 경험이 있는 전문기관에 의뢰하여 시행할 예정임.

- 검체 폐기 또는 보관의 방법적 안전성 및 윤리적,법적 타당성 : 제공받은 유전자원은 연구대상자들이 유전자검사 동의서에 명시한 목적에만 이용할 것이며, 보관기간이 경과한 DNA 시료는 『생명윤리 및 안전에 관한 법률』과 『폐기물관리법령』이 정하는 절차에 따라, 생물안전표시가 부착된 전용 폐기함에 담아, 전문처리업체에 위탁하여 폐기할 것임. 본 연구에서 사용될 유전자원 및 혈액시료들은 혈액시료 전용 냉장고에 보관되고 상기 과제를 수행하기 위한 목적으로만 사용될 것이며, 상업적 또는 비윤리적인 목적을 위한 수단으로는 사용되지 않을 것임.

○ 신체검진 및 혈액검사 소견에 대한 상담

- 신체측정치와 혈액검사, 영양평가 소견을 종합해서 의사, 임상영양전문가, 체육전문가의 1:1 대면상담을 시행할 예정임

- 대사적 합병증, 심리적 문제 동반 여부에 따른 전문적 치료 연계

: 혈액검사 및 혈압 측정에서 대사적 합병증이 동반되어 중재프로그램 참여 뿐 아니라 적극적인 치료가 필요한 경우에 대해서는 근처 전문기관으로의 진료를 의뢰하여 해당 질병에 대한 치료를 받게 조율할 예정임.

○ 연구진행에 관련된 모든 사항은 주관연구기관의 연구윤리심의위원회 (IRB) 심의를 받은 후 진행할 예정임

○ 기초자료 분석 및 단기효과 평가

- 단면적 역학분석, 중재효과 판정

: 각 변수간의 단변량 분석 (x2 분석, t-test) 후 변수의 성격에 따라 선형회귀 혹은 로지스틱 회귀분석을 시행하여 고도비만과 관련된 요인을 분석.

□ 연구개발 내용 : 2차 년도

○ 중재프로그램 지속 수행 (Phase 2&3)

- 강도 높은 1:1 중재 후 집단 유지 프로그램 시행

: 영양 및 운동 등을 주제로 한 집단 (한 단위는 약 30명 정도, 집단 프로그램 성격에 따라 전체 참여자가 될 수 있음) 중재 교육 시행

: 체중조절의 기본요소인 영양과 운동 이외에도 진로체험교육(다양한 직업을 가진 강사들이 와서 그 직업군에 대한 이해도를 높이는 체험교실)이나 연 1회 정도의 모든 참여자가 함께 하는 이벤트 시행

: 아이들 대상의 집단 교육 외에도 동시에 부모 대상의 교육을 함께 시행

- 월 별 주요 실천을 위한 주요 미션 수행 : Key message

: 월별 집단 유지 프로그램 시행 시 월 별로 Key message의 형태로 미션을 수행하고 이 주제를 업무 협약된 단체, 지자체, 혹은 보건기관과 함께 월별 캠페인으로 활용.

: 연구팀에서 운영하는 각종 형태의 SNS (블로그, 카페 등)와 단체 메시지방 등을 통해 월별 공지할 예정. 이는 공개된 자료이므로 국민 누구나 접근이 가능.

○ 이탈률 최소화를 위한 방안 마련

- 월 별 순응도 평가를 통해 탈락 요인을 파악하고 이를 개선하도록 노력

- 단체 프로그램에 부모 참여 프로그램을 운영 혹은 이벤트 데이를 운영하여 가족의 참여도를 높임

- 출석 및 각종 미션 수행에 대한 스탬프 확인을 통한 보상체계 활성화

○ 체중조절 추세 둔화 방지를 위한 1:1 Booster 중재 세션 진행

- 초기 6개월간의 강도 높은 1:1 중재 -> 6개월간의 집단 활동 -> 이후 1:1 집중 프로그램을 통한 생활습관 재평가 및 교정 시행

□ 연구개발 내용 : 3차 년도

○ 장기 중재 유지 프로그램 운영 (Phase 4)

- 집단 중재 교육 시행

- 집단 중재 교육 시 고도비만 소아청소년을 위한 내용과 부모님을 대상으로 한 내용이 동시에 진행됨. 집단활동을 통해 스트레스 대처법, 체중조절 시도에 있어 어려운 문제 해결법 등에 대해 함께 논의하는 기회를 가지게 됨.

- 치료자 면담 빈도 완화 (3개월 1회 -> 6개월 1회)

○ 장기적 효과 평가

중재 유형별, 동일 중재군 내 효과 비교

- 자료의 분석은 SPSS, STATA, 또는 SAS 통계 패키지를 이용하여 분석

- 중재 유형별 중재효과 판정

: 중재 후 효과판정을 위해서는 각 군별 전후 차이값을 산정하여 ANOVA 분석을 시행하거나 mixed model analysis를 시행하여 각 군 간의 효과비교분석.

- 동일 중재군 내에서 지표개선에 관련 있는 변수에 대한 분석

- 중재 기간에 따른 효과 비교

: 단기, 중기, 장기에 따른 효과를 비교 분석하여 장기간 유지프로그램의 단기 혹은 중기 프로그램에 대한 장점이 있는지를 판별

○ 자료 정리 및 검체 기탁

- iCReaT에 입력된 각종 측정 및 설문결과 데이터 재점검 통해 데이터의 정도 관리 및 정확도 확보

- 수집된 검체 국립중앙인체자원은행에 기탁 완료

○ 집담회 혹은 공청회를 통한 중재 프로그램 효과에 대한 공유

- 장기적 중재효과 및 고도비만 관련 역학자료 분석 결과 공유

- 시행된 중재프로그램의 향후 활용방안에 대한 논의

3) 개발된 프로토콜을 토대로 다양한 중재 프로그램 운영

- 전문의와 건강 상담, 1:1 영양상담, 신체활동증진 프로그램, 영양정보 공유 및 식단 조절 프로그램 등 전문가 풀 활용

4) 중재 프로그램 전, 후로 설문 및 검진 수행

- 신체계측, 혈압 (2회 이상 측정), 비만도 측정 (체성분검사, 허리둘레), 혈액 검사 (빈혈, 고지 혈증, 당뇨 및 간기능 검사, 공복 인슐린치 측정)영양조사 (3일간 식사일기), 설문조사 (생활습 관 및 신체활동 (GPAQ) ), 정신건강 (아동청소년 행동평가척도) 등

- 검사 결과 통보 및 사후관리

: 고도비만군

-> 검사 후 직접방문하여 1:1 맞춤형 결과 상담 및 처방 (영양처방 및 운동처방 포함)

-> 대사적 합병증 동반되어 보다 적극적인 치료가 필요한 경우는 주거지 인근 전문의료기관 으로 진료의뢰 조율

-> 일정기준 이하 저소득층 아동이 병원 진료가 필요한 경우, 사회사업팀과 연계하여 진료비 지원 예정

5) 중재효과 분석 및 유형별 효과 분석

- 중재 프로그램에 따른 신체계측, 생화학, 생활습관, 식이섭취 등의 변화 등의 분석 결과 확인- 중재 프로그램의 효용성 평가

4. 기대효과 및 활용방안

○ 역학자료 분석을 통한 고도비만 관련 요인 파악

- 보건정책수립 및 평가를 위한 기초자료를 제공

- 향후 고도비만 관련 예방 및 중재의 기초자료 활용 가능

○지역사회 기반 고도비만 관리 프로그램의 기본 모델 제시

- 향후 국가 차원에서 각 부처별 협력을 통한 고도비만 관리 방안을 구축하고 이를 전국 단위로 확산하는데 중요한 기본 모델로 활용이 될 수 있음

○장기적 추적관리에 적합한 모델 제시 가능

- 보다 체계적이고 조직적인 장기운영 가능한 레지스트리형 중재프로그램의 바람직한 기본 모델을 제시
