# Supplementary material for: Effects of circuit training or a nutritional intervention on body mass index and other cardiometabolic outcomes in children and adolescents with overweight or obesity
Source: PLoS One. 2021 Jan 28;16(1):e0245875. doi: 10.1371/journal.pone.0245875 (PMC7842905; doi:10.1371/journal.pone.0245875)
Supplement: S2 File — (DOCX) [file pone.0245875.s017.docx]

**S2 File.** Study protocol (English)

Long term follow-up intervention study of morbid obese children and adolescents (ICAAN)

Name of Study Site: Hallym University Medical Center

Contact Person for Principal Investigator: Prof. Kyung Hee Park

(beloved920@naver.com)

Source of Monetary/Material Support : Korea Centers for Disease Control and Prevention

1. Background and Purpose

1-1. Research and development overview

☐ Current status of morbid obesity in Korea

Over the past few decades, the prevalence of obesity and severe obesity in children and adolescents has increased worldwide. In Korea, according to the statistical analysis of the sample of student health examinations in 2015, the severe obesity rate of elementary, middle and high school students increased from 0.8% in 2007 to 1.6% in 2015.

☐ Characteristics of morbid obesity in childhood and adolescence

Obesity in children and adolescents is at high risk of transition to adult obesity. Whitaker et al. reported that 55% of obese children aged 6-9 years, especially 75% of adolescents aged 10-14 years, were obese in adulthood. Considering only severe obesity, 69% of 6-9 year-olds and 83% of 10-14 year-olds were obese in adulthood.

It has been reported that obesity in children and adolescents not only increases the incidence of metabolic syndrome and cardiovascular diseases such as hypertension, type 2 diabetes, dyslipidemia and arteriosclerosis in adulthood, but also increases the risk of cardiovascular disease in obese children and adolescents at an early age.

When compared to moderately obese children, children with severe obesity are at greater risk for adult obesity, early atherosclerosis, hypertension, type 2 diabetes, dyslipidemia, metabolic syndrome, obstructive sleep apnea syndrome, fatty liver disease and premature death. Severely obese children and adolescents had a threefold increased risk of metabolic syndrome with respect to moderately obese children and adolescents. Increasing gradation of obesity was associated with higher risk for hypertension, with a nearly three-fold increased risk when comparing severe to moderate obesity in children and adolescents. The Bogalusa Heart Study demonstrated that 39% of children with moderate obesity had at least two cardiovascular risk factors, while 59% of children with severe obesity had at least two cardiovascular risk factors.

Children and adolescents with obesity have higher rates of lower self-esteem (athletic/physical competence and physical appearance perceptions), lower physical functioning and lower social acceptance and functioning compared with non-overweight children and adolescents and severely obese children and adolescents have lower health-related quality of life than children and adolescents who are healthy and similar quality of life as those diagnosed as having cancer.

Studies on socioeconomic status and obesity in childhood and adolescents reported that the lower the incomes, the higher the prevalence of obesity. A review study reported that associations between socioeconomic status and adiposity in childhood and adolescents are predominantly inverse, and another study suggested that childhood and adolescents socioeconomic status affects obesity even in later life. A recent study found that community socioeconomic deprivation was associated with higher body mass index in childhood and adolescents and more rapid growth of body mass index over time.

2. Research Objectives

○ To develop the intervention method of morbid obesity suitable for Korean children and adolescents and to determine its effectiveness

○ Collection of health information of Korean children and adolescents with morbid obesity through a long-term follow-up study

○ To develop early recognition education programs for national health practice

3. Research contents

1) Research subjects

Age group: Elementary and middle school students

Criteria: ≥ 85th percentile of age- and sex-specific body mass index according to the 2007 Korean National Growth Charts

Definiton: Overweight was defined as a body mass index ≥ 85th percentile for age and sex, mild to moderate obesity was defined as body mass index ≥ 95th percentile for age and sex or ≥ 25 kg/m^2^, and severe obesity was defined as body mass index ≥ 35 kg/m^2^ or ≥ 120% of the 95th percentile.

Recruitment methods: online recruitment notice on websites and social networking services, television, newspapers, and flyers

2) Development and application of intervention protocols

Phases 1, 2, 3, and 4 comprise the intensive intervention (for 6 months), group activity (for 6 months), booster (for 3 months), and group activity (for 9 months), respectively.

All participants receive the usual care, including one-to-one medical consultation, workbook provision for goal setting and behavioral modification, exercise counseling, physical activity monitoring and feedback, and one-to-one nutritional counseling. In addition, risk factors related to obesity as well as factors related to obesity management are selected and named Mission 5, and all participants are asked to participate. The contents of Mission 5 were as follows: drink water instead of other drinks; exercise more than 1 hour per day; reduce screen time to less than 2 hours per day; eat 5 or more vegetables and fruits every day; get enough sleep for more than 8 hours per day. At the first visit, a doctor explains anthropometric measurements and laboratory test results and assessed health risks and lifestyle of all participants. Every 6 months, the doctor reviews the workbook and conducted one-to-one medical consultation. In addition to the medical consultation, all participants receive exercise counseling from an exercise specialist, which focused on increasing and decreasing physical activity and inactivity, respectively. One-to-one nutritional counseling is conducted monthly by a clinical dietitian for all participants during phase 1. Nutrition counseling is based on a balanced diet that included sufficient water intake, recommended caloric intake and balanced distribution of nutrients. The contents of the nutritional counseling comprised of 8 sessions of 25 minutes each during phase 1.

The participants assigned to the exercise group also receive the usual care during the intervention period, and additionally participate in the exercise programs weekly in the first 3 months and biweekly in the next 3 months during phase 1. The exercise program requires participants to exercise 3 days/week for 60 minutes/session (one group exercise session and two home-based exercise sessions) at 60% to 80% of the maximal heart rate. The group exercise program involves bodyweight circuit training, which consisted of 6 different exercises. Each exercise is performed for 1 minute with 30 seconds rest; the entire workout takes approximately 10 minutes to complete and is named the “ICAAN Exercise”. During the group exercise, participants wear a heart rate monitor to ensure appropriate exercise intensity. The home-based exercise is a repeat process of the group exercise. The goal is to perform 30 minutes of aerobic exercise, such as running and cycling in addition to the ICAAN Exercise for 30 minutes at home, more than twice weekly. For the home-based exercise, a daily exercise journal prepared by the exercise group is reviewed every week for the first 3 months, and every other week for the next 3 months.

The participants assigned to the nutritional group also receive the usual care during the intervention period. In addition, one-to-one customized nutritional counseling according to the Nutritional Care Process model is conducted monthly by a clinical dietitian during phase 1. The the Nutritional Care Process contains four distinct but interrelated steps: nutritional assessment, diagnosis, intervention, and monitoring / evaluation, which enables systematic and efficient nutritional counseling. The nutritional counseling is comprised of 8 sessions during phase 1. Each session is conducted for 25 minutes. The nutritional group is also provided with weekly nutritional feedback during phase 1.

3) Operate intervention programs based on the developed protocol

Professional counseling with health professionals, 1:1 nutrition counseling, physical activity promotion programs, nutrition information sharing and diet control programs

4) Questionnaires and measurements before and after the intervention program

Questionnaires completed by children and adolescents include dietary habits, physical activity (the Global Physical Activity Questionnaire), drinking, smoking, sleeping time, screen time (television / computer use), inactivity time, and mental health (depression, stress, etc.). Questionnaires completed by parents or caregivers include education, monthly household income, past medical history, marital status, and the child’s birth-related variables (birth weight, etc.).

Dietary intakes are collected using 3-day food records (2 weekdays and 1 weekend day), after which, a clinical dietitian double-checks the records using food models. The participant nutrient intakes are assessed using a computer-aided nutritional analysis program.

Body weight and composition are measured by Bioelectrical Impedance Analysis after a 10-hour fast and voiding, with the participant barefoot wearing lightweight indoor clothing. Height is measured by a stadiometer while the participant is barefoot. Weight and height are measured to the nearest 0.1 kg and 0.1 cm, respectively. Body mass index values (weight in kilograms divided by height in meters squared) are converted into percentiles and z-scores based on age- and sex-specific body mass index of the 2007 Korean National Growth Charts. Waist circumference is measured at midpoint between the last rib and the top of the iliac crest to the nearest 0.1 cm using a non-elastic tape measure. Parental measurement data (height, body weight, body composition, and waist circumference) are also collected. To assess body composition, an additional whole-body dual-energy X-ray absorptiometry scanner performs a series of transverse one-centimeter scans starting at the participant’s head and progressing toward the feet after a 10-hour fast and voiding. dual-energy X-ray absorptiometry assessments are made by a certified radiology technologist.

Blood pressure is measured twice in the right arm of the participants in the seated position using a digital automatic blood pressure monitor. Venous blood samples are obtained after 12 hours of fasting to determine the fasting plasma glucose, fasting plasma insulin, high-density lipoprotein cholesterol, low-density lipoprotein cholesterol, triglyceride, aspartate aminotransferase, alanine aminotransferase, gamma-glutamyl transferase, high-sensitivity C-reactive protein, and adiponectin. The homeostasis model assessment for insulin resistance is used to determine insulin sensitivity and is calculated using fasting plasma glucose and fasting plasma insulin.

Cardiorespiratory fitness is assessed via 3-minute YMCA step test. Prior to the test, the participants are asked to sit in a chair for 2 to 3 minutes of rest and instruction. During the test, the participants step (up-up, down-down) for 3 minutes on a 30 cm height step box. A metronome is used to ensure the step frequency (24 steps/minute) and sets at 96 beats/minute. Heart rate is recorded during the test and recovery phases. Post-exercise HR is measured for 1 minute after the end of the test. Muscular strength is assessed using 1-repetition maximal test before and after the intervention. Upper- and lower-body muscular strength are assessed using chest press and leg extension machines, respectively. Prior to the test, participants are instructed by the exercise physiologists on the proper lifting techniques and test procedure, as prescribed by the American College of Sports Medicine’s guidelines. Briefly, a perceived maximum weight is obtained from a participant following a warm-up of 5 to 10 repetitions at 40% to 60% of the perceived maximum weight and a subsequent test of 4 to 5 repetitions at 60% to 80% of their perceived maximum weight. Finally, a small amount of weight (2 to 5 kg) is added, and a 1- repetition maximal lift is attempted. If the lift is successful, the participant rests for about 3 minutes and then attempts to lift a heavier weight (additional 2 to 5 kg). If the lift is not successful, a small amount of weight is removed, and another 1- repetition maximal list is attempted. The goal of the procedure is to determine the 1- repetition maximum within 4 maximal attempts.

5) Analysis of intervention effects

The primary outcome of the study is the body mass index z-score (standardized using age- and sex-specific body mass index from the 2007 Korean National Growth Charts), and the percentage of the 95th percentile of age- and sex-specific body mass index. Secondary outcomes include body composition variables (body fat, lean body mass, etc.), cardiometabolic risk markers (blood pressure, fasting plasma glucose, fasting plasma insulin, homeostasis model assessment for insulin resistance, high-density lipoprotein cholesterol, low-density lipoprotein cholesterol, triglyceride, aspartate aminotransferase, alanine aminotransferase, gamma-glutamyl transferase, high-sensitivity C-reactive protein, adiponectin, etc.), nutrition (total energy intake), and cardiorespiratory fitness and muscular strength.

4. Expected effect and utilization plan

○ Identify factors related to morbid obesity through analysis of epidemiological data

-Provide basic data for health policy establishment and evaluation

- Provide basic data for prevention and intervention of morbid obesity

○ Provide a basic model of a community-based morbid obesity management program

- Propose a basic model for establishing a plan to manage morbid obesity through cooperation among ministries at the national level and spreading it across the country.

○ Provide a model suitable for long-term follow up

-Propose a basic model that is desirable for a more systematic and organized long-term registry-type intervention program
